# New Perspectives on the Associations between Blood Fatty Acids, Growth Parameters, and Cognitive Development in Global Child Populations

**DOI:** 10.3390/nu15081933

**Published:** 2023-04-17

**Authors:** Vanessa N. Cardino, Travis Goeden, William Yakah, Amara E. Ezeamama, Jenifer I. Fenton

**Affiliations:** 1 Department of Food Science and Human Nutrition, Michigan State University, East Lansing, MI 48824, USA; cardinov@msu.edu (V.N.C.); goedentr@msu.edu (T.G.); 2 Department of Pediatrics, Columbia University Medical Center, New York, NY 10032, USA; wy2381@cumc.columbia.edu; 3 Department of Psychiatry, Michigan State University, East Lansing, MI 48824, USA; ezeamama@msu.edu

**Keywords:** fatty acids, linoleic acid (LA), alpha-linolenic acid (ALA), highly unsaturated fatty acids (HUFAs), essential fatty acid deficiency (EFAD), malnutrition, growth, cognition

## Abstract

Malnutrition is prevalent in low-middle-income countries (LMICs), but it is usually clinically diagnosed through abnormal anthropometric parameters characteristic of protein energy malnutrition (PEM). In doing so, other contributors or byproducts of malnutrition, notably essential fatty acid deficiency (EFAD), are overlooked. Previous research performed mainly in high-income countries (HICs) shows that deficiencies in essential fatty acids (EFAs) and their *n*-3 and *n*-6 polyunsaturated fatty acid (PUFA) byproducts (also known as highly unsaturated fatty acids or HUFAs) lead to both abnormal linear growth and impaired cognitive development. These adverse developmental outcomes remain an important public health issue in LMICs. To identify EFAD before severe malnutrition develops, clinicians should perform blood fatty acid panels to measure levels of fatty acids associated with EFAD, notably Mead acid and HUFAs. This review demonstrates the importance of measuring endogenous fatty acid levels for measuring fatty acid intake in various child populations in LMICs. Featured topics include a comparison of fatty acid levels between global child populations, the relationships between growth and cognition and PUFAs and the possible mechanisms driving these relationships, and the potential importance of EFAD and HUFA scores as biomarkers of overall health and normal development.

## 1. Introduction

In 2021, 45.4 million children under 5 years old suffered from wasting, and 149.2 million children under 5 years old were stunted globally [1]. Although the global prevalence of malnutrition-related growth failures has decreased overall in the past two decades, it is concentrated within a small number of low-middle-income countries (LMICs) [2]. In 2020, 94% of stunted children and 97% of wasting children either lived in Asia or Africa [2]. This is especially alarming when considering that many of the LMICs within these continents are unlikely to achieve at least one of the three 2030 Sustainable Development Goals regarding child malnutrition [2].

Childhood malnutrition, as it occurs during a period of rapid growth, can lead to abnormal physical and cognitive development, which can ultimately lead to increased morbidity and mortality in both childhood and adulthood [3]. Specifically, undernutrition during childhood can result in a low weight-for-age z-score (WAZ), a standardized measure of underweight, a low height-for-age z-score (HAZ), a standardized measure of stunting that is largely correlated with cognitive deficits, and a low weight-for-height z-score (WHZ), a standardized measure of recent, life-threatening weight loss referred to as wasting [1,3]. Undernutrition and micronutrient-related malnutrition can also lead to poor cognitive skills, such as verbal and spatial ability, reading and scholastic ability, and neuropsychologic performance [4]. Considering the prevalence of childhood malnutrition and its adverse outcomes, there is an urgent need to focus malnutrition prevention and remediation efforts on child populations in LMICs.

Childhood malnutrition can develop due to environmental, behavioral, municipal, and biological factors [5,6,7]. The identification of community childhood malnutrition in LMICs, however, is largely dependent on the physical manifestation of malnutrition, which is synonymous with protein energy malnutrition (PEM) [8]. Thus, malnutrition is often assessed using three categories of anthropometric measurements: those which assess body mass (such as weight), those which assess linear growth (such as height), and those which assess body composition (such as skinfold thickness, mid-upper arm circumference, and the relative proportions of subcutaneous fat and muscle) [9]. This is problematic because the sole use of anthropometric measurements to diagnose malnutrition can undermine the complexity of the condition. The anthropometric diagnosis of PEM does not allow clinicians to measure less apparent symptoms characteristic of malnourished individuals, such as impaired cognitive development. Additionally, PEM takes time to develop and, therefore, diagnose, meaning that the affected individuals were likely dealing with malnutrition for months or even years [10]. Finally, the focus on protein deficiency may prevent clinicians and healthcare workers from identifying other nutritional deficiencies, which may lead to PEM and more severe symptoms.

One such nutritional deficiency is essential fatty acid deficiency or EFAD. EFAD is a deficiency in the essential fatty acids (EFAs) linoleic acid (LA, 18:2*n*-6) and α-linolenic acid (ALA, 18:3*n*-3). It is prevalent in both LMICs and high-income countries (HICs), likely a consequence of the Westernization of diets globally which results in higher saturated fatty acid (SFA) intake and lower polyunsaturated fatty acid (PUFA) intake [11,12,13]. Since the human body cannot synthesize EFAs, humans must obtain them through the diet via food sources such as nut and vegetable oils [14]. Desaturase enzymes, such as delta-5-desaturase (D5D) and delta-6-desaturase (D6D), are two enzymes that modify the conversion of LA and ALA into their downstream omega-6 (*n*-6) and omega-3 (*n*-3) PUFA products [15,16]. As the body becomes deficient in *n*-3 ALA and *n*-6 LA (the fatty acids (FAs) which have the highest affinity for desaturases), it stops synthesizing *n*-3 and *n*-6 byproducts such as arachidonic acid (ARA, 20:4*n*-6 or triene) and begins converting the precursor omega-9 (*n*-9) FA oleic acid into Mead acid (tetraene, 20:3*n*-9) [17]. The tetraene:triene (T:T) ratio is the gold standard for diagnosing EFAD, as a value greater than 0.02 signifies deficiency, while values greater than 0.2 represent severe deficiency [18,19]. Mead acid levels > 0.21% of total FA may also be used as a clinical cut-point for severe EFAD diagnosis, as increases in Mead acid are associated with low EFA intake in populations which did not meet the criteria for EFAD [17,18].

T:T ratio and Mead acid levels correspond with physical symptoms of severe EFAD such as weight loss, skin changes, and visual and cognitive impairment as well as change in organ function [20,21,22]. The similarity between these adverse outcomes caused by EFAD and those caused by PEM demonstrates that the presence of EFAD and PEM in an individual may compound to produce more severe functional disruptions [9,23]. EFAD exacerbates the effects of PEM, and PEM may induce more severe EFAD because it disrupts the digestion and absorption of free fatty acids [9,23]. Thus, clinicians should consider assessing both EFAD and PEM in potentially malnourished individuals.

Clinicians may also measure other EFA byproducts to further elucidate the mechanisms relating EFAD to abnormal growth and cognitive development. Such byproducts include the PUFAs dihomo-gamma-linolenic acid (DGLA, 20:3*n*-6), arachidonic acid (ARA, 20:4*n*-6), docosatetraenoic acid (DTA, 22:4*n*-6), docosapentaenoic acid (DPA, 22:5*n*-6 or *n*-3), eicosapentaenoic acid (EPA, 20:5*n*-3), and docosahexaenoic acid (DHA, 22:6*n*-3). The metabolism of EFAs into their PUFA byproducts is not efficient in mammals, so ARA, EPA, and DHA are considered “conditionally essential” [24]. Research shows that excess dietary intake of both ARA and DHA may prevent EFAD due to the retro conversion of these PUFAs into ALA and ARA [20]. EFA byproducts display other important biological functions; DHA and ARA are the main fatty acids found in brain tissue, and they are rapidly incorporated into neural membranes and the retina during childhood [25,26,27]. The relative amounts of these metabolites, also known as highly unsaturated fatty acids (HUFAs), can additionally influence the inflammatory state of the body and further lead to adverse physiological outcomes [28]. More specifically, a higher percentage of *n*-3 HUFAs, notably DHA and EPA, among total fatty acids is associated with cognitive improvement at levels up to 10% total FA [29,30]. From measuring levels of these various fatty acids to establish associations with growth and cognition in child populations around the globe, researchers can further study the underlying mechanisms which drive this association. Thus, the measurement of HUFAs can allow researchers and clinicians to better understand how EFAD contributed to malnutrition symptoms.

The purpose of this review is as follows: (1) use current fatty acid levels from various child populations across the globe to demonstrate the importance of measuring endogenous fatty acid levels to measure FA intake; (2) to provide a comprehensive overview of the research relating fatty acids to growth and cognition in children and elucidate the underlying mechanisms responsible for these relationships; (3) introduce the potential importance of HUFAs to normal growth and cognitive development in children; and (4) promote the measurement of a comprehensive fatty acid panel to determine specific dietary or supplementation interventions which can lower EFAD, PEM complications, and the likelihood of childhood developmental issues carrying on into adulthood.

## 2. Comparison of PUFA Levels between Global Child Populations

Studies that reported FA levels in child populations 16 years of age and under demonstrate that FA levels, specifically EFA levels, vary greatly (Table 1). When comparing EFA levels, child whole-blood LA levels range from a low of 8.83% total FA in Pakistan [31] to a high of 36.8% in Uganda [32,33], and child whole-blood ALA levels range from 0.17% in Pakistan [31] and 0.54% in the United Kingdom [34]. HUFA levels also demonstrated variability. ARA levels were highest among South Korean children (15.3%) [35] and lowest among Cambodian children (3.6%) [36]. Moreover, EPA levels varied from 0.1% in Cambodia [36] to 0.84% in Inuit children living in Canada [37], and DHA levels varied from 0.8% in Cambodian children [36] to 8.3% in South Korean children [35]. Child populations had amounts of DGLA as low as 0.56% in Uganda [32,33] and as high as 19.1 ng/uL in Gambia [38], and DTA levels as high as 3.05% in Pakistan [31] and as low as 0.018% in Uganda [32,33]. DPA*n*-6 levels ranged from 0.01% (in Uganda) [32,33] to 3.32 ng/uL (in Gambia) [38], while DPA*n*-3 levels ranged from 0.067% (in Uganda) [32,33] to 11.9 ng/uL (in Gambia) [38]. Mead acid, which was the most understudied fatty acid, ranged from 0.07% in Burkina Faso [39] to 0.37% in Pakistan [31]. Although these FA levels were measured from a variety of FA pools which may reflect long- or short-term dietary intake, including whole blood, red blood cells, plasma, and serum [40,41,42], research shows that the relative percentages of fatty acids are comparable regardless of sample type [43]. A closer examination comparing LMIC child populations to HIC child populations may explain these wide ranges.

In general, child populations in LMICs did not necessarily have lower levels of EFAs and their HUFA metabolites (Table 1). Our observations do not fully support prior evidence which shows that gross national income is significantly correlated to higher ARA and DHA intake [55] and that LMIC populations are at an increased risk of underconsumption of important dietary FAs [56,57]. For instance, Tanzanian infants and children had DGLA and DPA *n*-6 levels higher than those in European and Colombian children [34,45,49,51,53,54], and South African and Ghanian children had higher levels of ARA than children from the United Kingdom, Norway, and Colombia [34,45,46,47,52,58]. In fact, southern Ghanian children had higher DHA levels than many of the HIC child populations observed [34,45,47,49,51]. Consequently, LMIC child populations had the highest level of a number of fatty acids. LA levels were the highest among Ugandan children aged 6–10 years (mean (SD): 36.8 (0.35)% total FA) [32,33], DGLA and DPA*n*-6 levels were highest among 3–9-month-old Gambian children (DGLA: 19.1 (6.49) ng/uL; DPA*n*-6: 3.32 (1.24) ng/uL)) [53], and DTA levels were highest among 0–5-year-old Pakistani children (median (IQR): 3.05 (2.13–3.85)% total FA) [31]. Lastly, DPA*n*-3 levels were highest among Gambian children aged 3–9 months (mean (SD): 11.9 (3.82) ng/uL) [38]. The discrepancy between our findings and previous results is likely because these earlier studies measured FA intake solely using food fatty acid composition and consumption data. By not measuring blood fatty acid levels, these studies relied on more subjective measures of FA intake and thus may not have accurately measured PUFA intake.

Considering these data, LMIC child populations tend to have higher levels of *n*-6 HUFAs, and HIC child populations tend to have higher levels of *n*-3 HUFAs. Since a higher proportion of *n*-3 HUFAs is linked to better health outcomes [28], children in LMICs may be at a greater health disadvantage during a critical developmental period. Mead acid levels cannot be compared by country income status because it was only measured in LMICs, but 0–5-year-old Pakistani children had the highest concentration of Mead acid (median (IQR): 0.37 (0.14–1.32)% total FA) [31]. As Mead acid is synthesized in both the absence of LA and ALA, which serve as the precursors for *n*-6 and *n*-3 HUFAs, respectively, children in LMICs may have lower levels of both *n*-6 and *n*-3 HUFAs which are both necessary in sufficient amounts during childhood [17,28,29,30]. Collectively, this evidence demonstrates that LMICs may be at a higher risk of unhealthful HUFA balance and lower HUFA levels overall, but it is difficult to compare the potential for EFAD across countries since Mead acid is not commonly measured or reported in studies performed in HICs.

FA levels among child populations differ greatly within country income categories (Table 1). For instance, Ugandan children aged 6–10 years have higher LA levels (mean (SD): 36.8 (0.35)% total FA) but lower DGLA (0.56 (0.20)%), DPA*n*-6 (0.014 (0.001)%, ALA (0.26 (0.10)%), and DPA*n*-3 levels (0.067 (0.003)%) [32,33] compared to other LMIC child populations within sub-Saharan Africa and Asia. Colombian children 5–12 years old, however, had LA levels (30.3 (3.10)%) [45] comparable to those of the Ugandan children. Additionally, 2–6-year-old southern Ghanian children had mean EPA levels of 0.80% (SD: 0.35) [47], which approach those of well-nourished Inuit children (0.84 (0.16)%) [37], whose diet consists primarily of cold-water fish, and healthy European children [49]. In Burkina Faso, which shares a border with Ghana, children’s whole-blood EPA levels (median (IQR): 0.13 (0.10, 0.18)%) [39] trended lower than those in southern Ghana [47]. Based on this evidence, it is important to compare LMIC child populations with one another rather than grouping them all together.

HIC child populations also exhibit variability in FA levels (Table 1). The mean DHA level of children 7–9 years old from the United Kingdom was 1.9% (SD: 0.53) [34], which was relatively low compared to LMIC child populations, notably Nepalese children from 0 to 12 months old (mean: 4.9%, SD not reported) [50]. Many other HIC child populations, however, had higher DHA levels than those seen in the United Kingdom, notably Inuit Canadian children aged 11–53 months (mean (SD): 2.67 (1.52)%) [37], Dutch children aged 7–8 years (2.8 (0.7)%) [51], Norwegian children aged 1 year (6.00 (1.00)%) [58], and South Korean children aged 4–6 years (8.3 (1.3)%) [35]. Although ALA levels were generally higher among HIC populations compared to LMIC populations, Colombian children aged 5–12 years (0.49 (0.15)%) [45] and children from the United Kingdom aged 7–9 years (0.54 (0.25)%) [34] had mean ALA levels which trended higher than those of other HIC child populations. 1-year-old Norwegian children (12 (2)%) [58] and 11–53-month Canadian Inuit children (10.11 (0.37)%) [37] had the highest ARA levels of the HIC populations, but they were still comparable to most LMIC child population ARA levels. LA and EPA levels were relatively comparable across HIC populations, but LA levels were noticeably higher among 7–8-year-old Dutch children (23.2 (2.3)%) [51] and 5–12-year-old Colombian children (30.3 (3.10)%) [45], and EPA levels were noticeably higher among 11–53-month-old Canadian Inuit children (0.84 (0.61)%) [37] compared to other HIC populations. The same is true for DPA*n*-3, which was also noticeably higher among Inuit children (1.54 (0.69)%) [37]. Overall, HIC child populations demonstrated fewer variations in FA levels than LMIC child populations.

Other factors besides country income status may contribute more substantially to differences in FA levels across child populations. Comparison of various child populations by age shows that older children, i.e., children 6–16 years old, demonstrate higher levels of LA and ALA, but younger children, i.e., 0–6 years old, demonstrate higher levels of DGLA, DPA*n*-6, EPA, DPA*n*-3, DHA, and Mead acid (Table 1). Thus, the age of children in the population of interest may influence their FA levels. Additionally, dietary intake largely influences FA levels. For instance, food frequency questionnaires from a study conducted in northern and southern Ghana demonstrated that the latter population consumed more fish compared to their counterpart due to their proximity to the Gulf of Guinea, which was confirmed by higher *n*-3 and lower *n*-6 whole-blood FA levels [46,47,59].

Dietary intake can also influence FA levels indirectly by altering the activity of the desaturase enzymes involved in the conversion of LA and ALA into their downstream PUFA byproducts, i.e., delta-5-desaturase (D5D) and delta-6-desaturase (D6D). Six-week EPA fish oil supplementation increased D5D activity by 25% and decreased D6D activity by 17% (*p* < 0.0001) [15]. FA intake also influences the expression of D5D and D6D by altering fatty acid desaturase (FADS) genotypes [60]. A study conducted among Greenlandic Inuit showed that the population experienced gradual changes in FADS1 and FADS2 expression over generations due to high *n*-3 PUFA consumption, which led to increased D5D activity [61]. Besides influencing the level of PUFAs in the blood, FADS expression may have implications for chronic disease, as increased FADS2 expression is linked to adverse health outcomes such as obesity, type 2 diabetes, and high triglyceride scores [60,62]. Factors such as dietary intake and genotypes should be considered over country income status as important determinants of population FA levels and resulting morbidities.

Overall, there is variability in the FA profiles of children globally. It is difficult to generalize and say that HIC child populations have more sufficient FA levels overall compared to those from LMICs. There is a trend, however, that LMICs have higher *n*-6 HUFA levels than HICs, while HICs have higher *n*-3 HUFA levels than LMICs, which may give children in HICs a health advantage. More researchers should consider measuring Mead acid levels in HICs populations to aid in comparison with levels of LMICs and determine if they have a similar prevalence of EFAD. Nevertheless, country income status may not be the best predictor of FA levels. Better determinants of FA levels include age, dietary intake, and genetics. To confirm these findings, more studies assessing the relationship between food consumption and FA levels in child populations of various age ranges are needed. Other studies should also be carried out to examine the effect of intake of certain fatty acids on epigenetics, notably the expression of FADS genotypes and resulting desaturase activity.

## 3. PUFAs and Growth Parameters

### 3.1. Associations between PUFAs and Growth Outcomes in Children

Research in recent years has attempted to demonstrate the association between PUFAs and growth parameters in children <16 years old (Table 2). During early development, EFAs are converted into their PUFA byproducts, which are critical for organogenesis, overall regulation of growth by providing structural integrity to cell membranes, growth hormone synthesis, and immune system function [63,64,65]. Thus, insufficient PUFA levels during periods of rapid growth, likely due to inadequate nutrition during infancy and childhood, may lead to irreversible growth abnormalities. Specifically, low overall PUFA levels may lead to stunting and wasting, while an imbalance of PUFA levels, characterized by a high *n*-6:*n*-3 ratio, may lead to obesity.

Deficiencies in PUFA before 16 years of age are linked to abnormal growth parameters. Commonly measured growth parameters include height-for-age z-score (HAZ) as a measure of stunting; weight-for-age z-score (WAZ), weight-for-height z-score (WHZ), and mid-upper arm circumference (MUAC) as measures of wasting and moderate acute malnutrition (MAM); and BMI-for-age z-score (BAZ) as a measure of weight category. Inadequate PUFA levels and growth parameters in infants are most common in LMICs, where the intake of food products high in both EFAs and PUFAs is low, and malnutrition and inflammation are high [32,36,39,45,50,66]. As PUFAs have been shown to resolve inflammation in young children, low PUFA intake likely exacerbates chronic inflammation resulting from malnutrition and environmental factors, which together disrupt normal growth [67].

The compounded effects of low dietary PUFA intake and acute malnutrition on growth in young children can be seen in LMICs. Jumbe et al. observed that the total *n*-9 PUFA levels and T:T ratios were negatively associated with HAZ but positively associated with BAZ and WHZ, while total *n*-6 PUFA levels were positively associated with HAZ (notably LA) but negatively associated with BAZ and WHZ (notably ARA) in 2–6-year-old Tanzanian children (all *p* < 0.05) [68]. In a study of northern Ghanian children of the same age, Adjepong et al. found that after adjusting for hemoglobin, HAZ and WAZ were positively associated with whole-blood ARA, DGLA, DTA, and the ratio of DGLA/LA (all *p* < 0.05) [59]. Collectively, these results suggest that *n*-6 PUFAs, specifically EFA LA, are beneficial for linear growth, while an increased *n*-9 PUFAs and an accumulation of Mead acid may prevent underweight but promote stunting, leading to larger weight-to-height ratios and BMIs.

A cross-sectional study of 668 school-aged children in Colombia, a country where the incidence of pediatric obesity has increased over the past few decades, showed that children with the highest ALA serum levels exhibited smaller BAZ increases than those with the lowest ALA serum levels (β = −0.45, 95% CI: −0.83, −0.07) from age 6 to age 14 [45]. These results propose that sufficient ALA levels during childhood may help to promote linear growth, resulting in healthy BMIs. Another study showed that 62% of a Malawian study population aged 1–5 years were stunted (HAZ < −2), and HAZ was associated with low serum DHA and ARA serum levels (*p* < 0.05), further supporting the significance of both *n*-3 and *n*-6 PUFAs in inhibiting stunting [66].

**Table 2:** Associations between blood fatty acid levels and growth outcomes among child populations in different countries and regions.

Country	Study Size	Age	Design	Intervention	Tissue	Growth Outcome Measured	Fatty Acids Measured	Main Outcomes	Study
Burkina Faso	1609	6–23 months	Cross sectional	-	Whole blood	MUAC, WHZ, HAZ	*n*-3 and *n*-6 PUFAs	Children with MAM had low levels of total PUFA, particularly *n*-3 PUFA Children diagnosed with MAM based on MUAC only had lower ARA levels than those diagnosed with MAM based on MUAC + WHZ or WHZ only	[39]
Cambodia	174	4 months–16 years	Cross sectional	-	Whole blood	HAZ, WAZ, BAZ, MUAC	SFA, MUFA, *n*-6 and *n*-3 PUFAs, T:T ratio, DPA*n*-6:DHA, *n*-6:*n*-3 ratio	HAZ was positively associated with total PUFA, LA, ARA, and total *n*-6 PUFA Height was positively associated with DHA and total *n*-3 PUFA	[36]
Colombia	668	5–12 years	Cross sectional	-	Serum	BAZ	*n*-3 and *n*-6 PUFAs, *n*-6:*n*-3 ratio	ALA was negatively associated with BAZ	[45]
Gambia	50	3–9 months	RCT	200 mg DHA and 300 mg EPA or 2 mL olive oil/day for 6 months	Plasma	MUAC, HC, skinfold thickness	*n*-3 and *n*-6 PUFAs	PUFA supplementation increased MUAC at 9 and 12 months and increased skinfold thickness at 12 months	[38]
Ghana (north)	307	2–6 years	Cross sectional	-	Whole blood	HAZ, BAZ, WAZ, WHZ	*n*-3, *n*-6, and *n*-9 PUFAs, T:T ratio, GLA:ARA, EDA:LA, DGLA:LA, ARA:DGLA	HAZ and WAZ were positively associated with ARA, DGLA, DTA, and DGLA/LA	[59]
Ghana (south)	209	2–6 years	Cross sectional	-	Whole blood	HAZ, WAZ	*n*-3 and *n*-6 PUFAs	No relation between essential fatty acids and other fatty acids and growth	[47]
Greece and Netherlands	529	4–6 years	Longitudinal	-	Blood PLs	WAZ, BAZ	*n*-3 and *n*-6 PUFAs, *n*-6:*n*-3 ratio	No association between cord blood PUFA levels and rapid infant growth nor BMI at 4–6 years of age	[69]
India	598	6–10 years	RCT	900 mg ALA + 100 mg DHA or 140 mg ALA/day for 12 months	Whole blood	HAZ, WAZ, MUAC	Total *n*-3 FAs, ALA, EPA, DHA	*n*-3 FA supplementation did not affect growth	[70]
Malawi	400	12–59 months	Cross sectional	-	Serum PL	HAZ	DHA and ARA	HAZ was positively associated with serum phospholipid DHA and ARA levels	[66]
Mexico	193	12–30 months	RCT	~1240 mg LA + 156 mg ALA + 33 mg DHA/day for 4 months	Serum	HAZ, WAZ, BAZ at 2 months and 4 months	SFAs, *n*-3 and *n*-6 PUFAs	Supplementation significantly increased ALA and DHA serum levels Supplementation did not significantly improve growth parameters	[71]
Nepal	303	4–10 months	Cross sectional	-	RBC	HAZ, WAZ	SFA, MUFA, *n*-6 and *n*-3 PUFAs, *n*-6:*n*-3 ratio, Omega 3 Index	HAZ was positively associated with DHA and ARA levels	[50]
Tanzania	334	2–6 years	Cross sectional	-	Whole blood	HAZ, WAZ, BAZ	*n*-3, *n*-6, and *n*-9 PUFAs, T:T ratio	HAZ was positively associated with LA and total *n*-6 PUFAs and negatively associated with Mead acid, T:T ratio, and total *n*-9 PUFAs BAZ was positively associated with oleic acid, total *n*-9 PUFAs, and T:T ratio and negatively associated with ARA and total *n*-6 PUFAs Weight-for-height z-score was positively associated with oleic acid and total *n*-9 PUFAs and negatively associated with ARA and total *n*-6 PUFAs	[68]
Uganda	240	6–10 years	Cross sectional	-	Serum	HAZ	SFA, MUFA, *n*-6 and *n*-3 PUFAs, T:T ratio, *n*-6:*n*-3 ratio, HUFA score	Total SFAs were negatively associated and total *n*-6 PUFAs were positively associated with HAZ regardless of HIV status Total MUFAs were negatively associated with WAZ regardless of HIV status Children with lowest DHA and LA levels had significantly lower WAZ Children with lowest DHA, total *n*-3 LPUFAs, and LA had significantly lower HAZ	[32]
Uganda	237	6–10 years	Longitudinal	-	Serum	HAZ	SFA, MUFA, LA, *n*-6 and *n*-3 PUFAs, Mead acid, T:T ratio, *n*-6:*n*-3 ratio	SFA levels were inversely associated with HAZ over 12 months Low vs. high LA, total *n*-6, and total PUFA levels predicted low HAZ over 12 months Low v. high levels of Mead acid and T:T ratio were each associated with high HAZ over 12 months	[33]
United States	27	0–6 months	Longitudinal	-	Plasma	HAZ, WAZ, BAZ	SFA, MUFA, PUFA, *n*-6:*n*-3 ratio	Low *n*-3 PUFAs and high SFA levels at birth were positively associated with high skinfold thickness at birth and an increase in BAZ from birth to 6 months	[72]
United States	348	10–16 months	RCT	200 mg DHA + 200 mg ARA via powder added to milk for 180 days	RBC	LAZ, WAZ, HC, MUAC, skinfold thickness, BAZ	*n*-3 and *n*-6 PUFAs, *n*-6:*n*-3 ratio	DHA + ARA supplementation did not influence growth or adiposity outcomes	[73]

HAZ = height-for-age Z-score; WAZ = weight-for-age Z-score; BAZ = BMI-for-age Z-score; MUAC = mid-upper arm circumference; HC = head circumference; LAZ = length-for-age Z-score; RCT = randomized controlled trial; PL = phospholipid; RBC = red blood cell; SFA = saturated fatty acid; MUFA = monounsaturated fatty acid, PUFA = polyunsaturated fatty acid; LA = linoleic acid; ALA = α-linolenic acid; GLA = gamma-linolenic acid; EDA = eicosadienoic acid; DGLA = dihomo-gamma-linolenic acid; EPA = eicosapentaenoic acid; DHA = docosahexaenoic acid.

Furthermore, within a cohort of 174 acutely malnourished Cambodian children aged 6 months to 16 years with an average T:T ratio of 0.02 (SD: 0.02) and an average total PUFA percentage of 20.1% (SD: 3.7%), Sigh et al. found positive associations between HAZ and mid-upper arm muscle area and whole-blood *n*-6 PUFA levels, including total *n*-6 PUFAs, LA, and ARA (all *p* < 0.05) [36]. This study also reported positive associations between height and DHA and total *n*-3 PUFAs (both *p* < 0.05). The high T:T ratio and low PUFA levels in this malnourished population potentially demonstrate how EFAD can compound the effects of PEM on linear growth.

A study of 1609 children aged 6–23 months from Burkina Faso further supports this hypothesis, as children diagnosed with MAM had low PUFA levels (mean (SD): 28.52 (2.78)% total FA), particularly low *n*-3 PUFA levels (mean (SD): 2.48 (0.65)% total FA) [39]. It is also interesting to note that in this same study, children diagnosed with MAM on the basis of MUAC only had significantly lower whole-blood ARA and EPA levels compared to those diagnosed with MAM on the basis of WHZ only (ARA: 6.88 v. 7.19% total FA, *p* = 0.007; EPA: 0.12 v. 0.14% total FA, *p* = 0.040), but higher whole-blood ALA levels (ALA: 0.22 v. 0.20% total FA, *p* = 0.042) [39]. A potential explanation for these results is that children with low MUAC may also be more likely to have decreased D6D activity, which may make them more prone to EFAD [39]. Thus, MUAC may be a valuable anthropometric measure for screening EFAD. Collectively, these studies support the positive relationship between low PUFA levels, high malnutrition, and abnormal growth in malnourished LMICs.

Similar associations between PUFA levels and growth parameters in well-nourished LMICs and HICs demonstrate that FA levels can still lead to abnormal growth parameters despite the absence of diagnosed PEM. Jain et al. observed that HAZ scores significantly increased by 0.16 (SD: 0.07) for every standard deviation increase in total *n*-6 PUFA serum levels regardless of HIV exposure in a cross-sectional study of 6-to-10-year-old Ugandan children (*p* = 0.035) [32]. They also found that higher levels of DHA and LA were associated with higher WAZ (both *p* < 0.029), higher levels of these fatty acids and total *n*-3 PUFAs were associated with higher HAZ (all *p* < 0.038), and the T:T ratio in this population was inversely associated with HAZ, WAZ, and BAZ (all *p* < 0.039) [32]. A longitudinal analysis of this same cohort strengthened these results by confirming that high total PUFA, total *n*-6 PUFAs, and LA levels and low Mead acid levels and T:T ratio predicted high HAZ over 12 months (all *p* < 0.05) [33].

Furthermore, a separate study of 303 well-nourished breastfed infants in Nepal, who exhibited total PUFA levels of 38% total FA, showed that HAZ was positively associated with DHA and ARA status (*p* = 0.04 and *p* = 0.002, respectively) [50]. A longitudinal study of American infants from birth to 6 months of age found that low neonatal plasma *n*-3 PUFA levels at birth were positively associated with adiposity-related growth parameters during development, i.e., high skinfold thickness at birth and increased BAZ from birth to 6 months of age (*p* = 0.046) [72]. Finally, a randomized controlled trial supplementing 3–9-month-old Gambian infants with 200 mg DHA and 300 mg EPA for 6 months showed that supplementation significantly increased MUAC at 9 months and 12 months and skinfold thickness at 12 months compared to the control group (all *p* < 0.022) [38]. Despite the fact that these populations were not malnourished, they still demonstrated a positive association between low FA levels and abnormal growth parameters, which demonstrates that FA deficiency can still lead to developmental issues in the absence of malnutrition.

Although many cross-sectional studies demonstrate a clear association between PUFA levels and growth parameters in both LMICs and HICs, some observational studies have not supported this association. A cross-sectional study of 2-to-6-year-old children in southern Ghana showed no association between whole-blood fatty acid levels and growth parameters (all *p* > 0.05) [47]. However, this study found that 93% of participants had eaten fish within 24 hours of data collection, which reflects the high consumption of seafood in southern Ghana due to its proximity to the Gulf of Guinea [47]. Since most of the children in this study have likely eaten a seafood-rich diet which promotes healthy growth from a young age [74], the incidence of stunting may not have been high enough (~10%) to be able to observe significant associations between fatty acid levels and abnormal growth. A longitudinal study of 529 4–6-year-old children from the Netherlands and Greece showed no association between cord blood phospholipid PUFA levels and rapid infant growth or childhood BMI (all *p* > 0.05) [69], which could be because cord blood FA levels may not translate to infant nor childhood levels once they are introduced to complementary foods. In conclusion, studies which did not support the association between PUFAs and growth parameters may have limitations that did not allow researchers to assess this association.

Many randomized controlled trials in both LMICs and HICs which supplemented children with *n*-3 PUFAs did not demonstrate any effects of *n*-3 PUFAs on growth. A randomized, double-blinded controlled trial of 6-to-10-year-old marginally malnourished Indian children showed no effect of *n*-3 FA supplementation (900 mg ALA and 100 mg DHA v. 100 mg ALA) paired with low or high micronutrient supplementation for 12 months on WAZ, HAZ, nor MUAC (all *p* > 0.05) despite significant increases in whole-blood *n*-3 levels (all *p* < 0.05) [70]. However, there was no true placebo group in this trial, as the “control” children were still receiving ALA supplementation, which may have been enough to promote normal growth; this hypothesis is supported by the results, as the overall prevalence of stunting/overweight decreased significantly from baseline (*p* < 0.001) [70]. A randomized controlled trial of 348 American preterm infants aged 10–16 months demonstrated that 200 mg DHA + 200 mg ARA supplementation for 180 days did not significantly improve any growth outcomes post-intervention (all *p* > 0.10) [73]. The lack of an effect in this study could be due to the fact that these preterm infants had a wide range of birthweights and gestational ages, which could make it more difficult to detect the effect of supplementation, or the supplementation was not long enough to effect short-term growth or adiposity [73]. Finally, a randomized controlled trial among 12–30-month-old Mexican infants demonstrated that supplementation of formula with ~1240 mg LA + 156 mg ALA + 33 mg DHA/day for 4 months did not significantly improve health outcomes (all *p* > 0.05) despite significantly increasing ALA and DHA serum levels (both *p* < 0.05) [71]. This study may not have seen an association because relative to other studies discussed [38], the amount of DHA supplemented was quite low [71]. Despite these limitations, these studies demonstrate that *n*-3 PUFAs may not affect growth outcomes in childhood as much as other FAs, notably *n*-6 PUFAs.

Overall, studies from both LMICs and HICs demonstrate a relationship between various fatty acid levels and growth parameters, regardless of malnutrition status in children ≤16 years of age. Notably, increases in both *n*-3 and *n*-6 PUFAs, including LA, ALA, and their metabolites, were related to normal HAZ, WAZ, and BAZ, which are the anthropometric measures often used to diagnose PEM. Randomized controlled trials studying FA supplementation and growth outcomes in childhood have shown overall null effects, but this may be because most of them prioritized studying *n*-3 PUFAs over *n*-6 PUFAs. Since both *n*-3 and *n*-6 PUFAs are important for growth [63,64,65], more research should be carried out specifically to study how growth outcomes may be affected by both *n*-3 and *n*-6 PUFA supplementation. Nevertheless, the existence of the association between PUFAs and growth in well-nourished populations demonstrates that children without malnutrition can still experience abnormal growth due to EFAD and deficiencies in EFA metabolites. However, more research can be conducted to monitor EFAD by including the measurement of LA, ALA, and Mead acid and the calculation of the T:T ratio. The comparison of these FA levels with growth outcomes will provide a more explicit picture of how EFAD may impact growth. Thus, the research supports the measurement of a large FA panel in both malnourished and well-nourished child populations to identify those most at-risk for poor growth so other researchers could provide targeted dietary alterations or supplementation initiatives to ensure that children are growing normally for their age into adulthood.

### 3.2. Potential Mechanisms Relating PUFAs and Growth Outcomes in Children

There are many mechanisms which may link growth parameters to PUFA intake, including gene transcription regulation and hormone production regulation. PUFAs likely affect DNA methylation through their role in one-carbon metabolism as methyl group donors and acceptors [75]. This effect is heightened during pregnancy, which is known to be a time of great epigenetic remodeling [76]. Additionally, insufficient methylation of certain gene variants linked to PUFA metabolism can further disrupt PUFA levels, which can eventually lead to alterations in growth during childhood [77].

PUFA levels directly impact the production of insulin-like growth factor 2 (IGF2), a hormone which regulates intrauterine growth [78]. Among a cohort of 973 Mexican pregnant women, infants of those supplemented with 400 mg DHA daily who were overweight or had preterm births demonstrated hypermethylation (a methylation proportion higher than the expected 50%) within the IGF2 P3 region of cord blood cells (*p* = 0.04) [79]. Additionally, the infants of supplemented mothers of normal weight experienced hypomethylation (a methylation proportion lower than the expected 50%) within the H19 differentially methylated region (DMR) of cord blood cells (*p* = 0.01) [79]. These results are significant because they demonstrate epigenetic differences between infants predisposed to or who quickly demonstrate abnormal growth and infants demonstrating normal growth.

Endogenous FA levels may also be influenced by the genetic code itself. Another cohort study demonstrated that variants in the genes which encode D5D and D6D, FADS1, FADS2, and FADS3, may affect gestation duration and birth weight [8]. Bernard et al. observed a significant association between plasma *n*-6 PUFA levels and maternal minor FADS1 and FADS2 alleles; those mothers who possessed the minor FADS1 variant rs174546 had higher plasma *n*-6 PUFA levels, while those who possessed the minor FADS3 variant rs174450 allele had lower plasma *n*-6 PUFA levels (*p* ≤ 0.001) [8]. Moreover, offspring FADS1 variant rs174546 was associated with a gestation duration of 1.7 days longer per each additional copy of minor allele G, and offspring FADS1 variant rs174450 was associated with a gestation duration of 1.9 days longer [8]. The significant associations between FADS1 and FADS3 genes and birth length and weight were removed when controlling for gestation duration [8], but the positive effects of certain FADS1 and FADS3 variants on gestation duration are likely due to circulating plasma *n*-6 PUFA levels. The lack of association between variants and *n*-3 PUFA levels is because dietary intake influences *n*-3 PUFA levels more than the FADS variant, and this study was not powered to detect the influence of the FADS variant on DHA levels [8]. Collectively, these results emphasize the synergistic effects of genetics and epigenetics on endogenous FA levels and consequently growth.

PUFAs may also affect growth by regulating the transcription of genes directly involved in fat deposition. A murine randomized controlled trial showed that a high fat, *n*-3 PUFA-rich diet (*n*-6:*n*-3 ratio of 0.84) decreased gene expression of compounds involved in lipogenesis, namely acetyl-CoA carboxylases (ACC1_2), and increased the gene expression of compounds involved in fatty acid oxidation, including carnitine palmitoyl transferase 1a (CPT1A) and acyl-coenzyme A oxidase 1 (ACOX1), in both the small intestine and the liver compared to a high *n*-6 PUFA diet (*n*-6:*n*-3 ratio of 13.5) [80]. This study also showed that a high-fat, *n*-3 PUFA-rich diet increased the number of macrophage clusters in adipose tissues [80], suggesting an anti-inflammatory effect. When considering these results with those of similar animal studies [14,81,82], ALA and its *n*-3 PUFA metabolites may help promote balanced lipid metabolism to yield appropriate BAZ scores and reduce adiposity-induced inflammation in young children, particularly those with a heightened *n*-6:*n*-3 ratio.

Additionally, ALA may further promote normal growth by regulating bone formation via insulin growth factor 1 (IGF1), a hormone responsible for stimulating adipogenesis and bone mineralization [83]. Mouse models have demonstrated that a low LA:ALA ratio (ranging from 1:1 to 5:1) in mice was shown to not only minimize adipocyte formation but maximize osteoblast formation as well by optimizing IGF1 levels [84]. Specifically, ALA affects IGF-1 production via its metabolites, EPA and DHA, which activate a peroxisome proliferator-activated receptor-α pathway [85]. Moreover, EPA and DHA decrease prostaglandin E2 (PGE2) in osteoblasts which inhibits adipocyte differentiation [86]. The synergistic production of IGF1 and PGE2 suggests a regulatory response to adiposity-induced inflammation, which further contributes to *n*-3 PUFA’s anti-inflammatory properties. These results have been confirmed by observational studies in pigs [87] and malnourished children [88] as well as randomized controlled trials performed on mice [89], post-menopausal women [90], and men with cardiovascular disease [91].

One randomized controlled trial in preterm infants showed daily DHA and ARA supplementation of 32 and 31 mg, respectively, during the first nine weeks of life significantly increased WAZ up to one year of age but decreased levels of IGF-1 and had no significant effect on growth parameters at eight years of age [92]. This may be explained by the lack of randomization based on growth (there were significantly more obese children in the control group); it is reasonable to believe that non-obese children would have lower IGF-1 levels than obese children given its positive effect on adiposity. These results could also be due to the inability to control for the standard Norwegian diet, which is rich in *n*-3 PUFAs such as DHA, suggesting that the amount that was supplemented to the intervention group would yield negligible differences. To further elucidate the relationship between PUFAs, IGF1, and growth in childhood, more randomized controlled trials must be performed in young children in various countries with differing diets.

Collectively, cohort studies and randomized controlled trials analyzing the relationship between EFA levels and growth have demonstrated that deficient EFA levels are associated with below-average growth parameters, regardless of country income or malnutrition status. Studies which did not observe a significant association between EFAs and growth in child populations may not have accounted for an increase in protein and energy intake in certain children or measured PUFA levels too early, which would limit the ability to detect an association [47,69,70]. Translational studies using animal models revealed mechanisms which demonstrate the importance of PUFAs in regulating growth through methylation of specific genes involved in fat metabolism, bone development, and inflammation. Observational studies and randomized controlled trials confirmed these findings. Future studies should consider potential confounders that may also affect growth, notably malnutrition, inflammation, and saturated fatty acid levels.

## 4. PUFAs and Cognitive Development

### 4.1. Associations between PUFAs and Cognitive Outcomes in Children

Researchers have also been interested in the association between PUFA levels and cognitive development in early and late childhood (Table 3). This interest stems from the relatively high concentrations of the ALA metabolites, EPA and DHA, in the hippocampus and prefrontal cortex [93,94]. The hippocampus and prefrontal cortex control cognitive functioning, including executive function, and undergo significant growth during childhood and early adolescence [95,96,97,98]. So, increased concentrations of these fatty acids in these parts of the brain may improve cognitive development in childhood and have long-lasting effects on cognitive functioning throughout life.

Cross-sectional studies of child populations in LMICs with insufficient fatty acid intake and whole blood fatty acid levels have supported the association between fatty acid intake and cognition. Adjepong et al. observed dimensional change card sort (DCCS) task performance, a measure of executive function, in 2–6-year-old children in northern Ghana [46]. Their results showed significantly positive associations between DCCS task performance and DHA and ALA levels (*p* = 0.02 and 0.01, respectively), and children with the highest levels of DHA, DHA + EPA, and total *n*-3 PUFAs were significantly more likely to complete at least one aspect of the DCCS task (*p* = 0.008, 0.047, and 0.02, respectively) [46]. Jumbe et al. determined LA was positively associated and ALA was negatively associated with DCCS performance in 4–6-year-old Tanzanian children [54]. The authors also found that within this population, children with a T:T ratio > 0.02 were four times less likely to complete the full overlap post-switch DCCS task, i.e., the task which requires the highest level of executive functioning (OR: 3.8; 95% CI: 1.05, 13.9) [54]. The discrepancy between the results of these two papers, specifically regarding the relationship between ALA and DCCS performance, suggests that more observational studies which measure Mead acid and calculate the T:T ratio are necessary to better elucidate the association between EFAD and cognitive outcomes.

Randomized controlled trials performed in African countries with low levels of fatty acid intake have also demonstrated a positive effect of fatty acid intake, specifically DHA supplementation, on cognitive function. Al-Ghannami et al. supplemented 9–10-year-old primary school students in Muscat, Oman with either 403 mg DHA/weekday from a fish oil supplement or 150–200 mg DHA/weekday from grilled fish for 12 weeks [103]. Both forms of DHA supplementation showed a significant increase in both EPA and DHA erythrocyte concentrations, but the fish oil supplement resulted in a significantly higher increase in these concentrations than the grilled fish (72% increase compared to 64% increase, *p* = 0.014) [103]. Additionally, only fish oil supplementation yielded a significantly positive effect on executive functioning measured using Part B of the Trail Making Test (*p* = 0.005) [103]. Dalton et al. gave 7–9-year-old primary school students of low socioeconomic status in South Africa 192 mg DHA and 82 mg EPA/weekday through fish spread for 6 months [52]. The supplemental group exhibited significantly higher EPA and DHA plasma and RBC concentration increases than the control group (all *p* < 0.0001), and supplementation resulted in higher recognition, discrimination index, and spelling test scores compared to children who were not supplemented (all *p* < 0.05) [52]. A more recent randomized controlled trial among 6–59-month-old Malawian children with severe acute malnutrition showed that the addition of DHA or oleic acid to ready-to-use-therapeutic food (RUTF) for four weeks significantly increased plasma phospholipid EPA and ALA levels and improved gross motor and social domain scores of the Malawian Developmental Assessment Tool (MDAT) compared to those who received standard RUTF (all *p* < 0.001) [101]. Overall, research from randomized controlled trials in LMICs demonstrates the beneficial effect of PUFA supplementation on cognitive outcomes.

Recent studies performed in Europe have shown that fatty acid levels may also affect cognitive function in well-nourished HICs. Montgomery et al. observed that 88.2% of their cohort of 7–9-year-old schoolchildren consumed less than the dietary recommendation of two servings of fatty fish per week and that their cohort had average whole-blood EPA and DHA levels (mean (SD): 0.56 (0.01)% and 1.9 (0.53)%) [34] similar to those observed in LMICs [46,52,54,103]. The researchers further observed that reading scores were positively associated with whole-blood DHA, EPA, DPA, DHA+EPA, and total *n*-3 PUFAs, while working memory was positively associated with DHA, EPA, DPA, DHA+EPA, and total *n*-6 PUFAs (all *p* < 0.05) [34]. Moreover, Oyen et al. provided daily servings of fatty fish containing roughly 1000 mg EPA + DHA or red meat to Norwegian Kindergarten students with a relatively high baseline erythrocyte EPA+DHA (known as the Omega-3 Index) of ~8% for four months [102]. The intent-to-treat analysis demonstrated that this supplementation did not improve Wechsler Preschool and Primary Scale of Intelligence (WPPSI-III) compared to red meat supplementation (*p* = 0.97) despite significantly higher erythrocyte EPA, DHA, and Omega-3 Index increases within the fish group (*p* < 0.0001) [102]. These results were supported by a longitudinal study of 7-year-old Dutch children which showed that DHA and ARA levels at birth and 7 years of age were not associated with cognition measured at 7 years using the Kaufman Assessment Battery for Children (*p* = 0.29 and 0.60, respectively) [51]. A randomized controlled trial of 7–12-year-old Chinese school children similarly demonstrated that 800 mg DHA/day for 6 months did not improve executive function measured via the Digit Span Backwards and Wisconsin card sorting test (all *p* < 0.08) despite significantly increasing ARA and DHA erythrocyte fatty acid levels (both *p* < 0.001) [44]. The null effect of these high dosages of DHA on cognitive function in Norway and China and the significant effect of EPA and DHA on cognitive function in the United Kingdom may be due to differing baseline DHA levels, i.e., sufficiency versus insufficiency, as Norwegian and Chinese children may receive adequate dietary fatty acids while English children may not. When looking at differences in baseline DHA levels, Norwegian (mean (SD): 6.46 (1.20)%) [102] and Chinese children (2.75 (0.59)%) [44] do in fact have higher levels than English children (1.9 (0.53)%) [34]. These studies provide evidence for a dose-response relationship between PUFA levels and cognitive outcomes that may plateau once PUFA sufficiency is achieved.

A randomized controlled trial carried out in Australia and Indonesia further demonstrated that PUFA supplementation affected well-nourished and malnourished children equally due to similarly high baseline PUFA levels [100]. Osendarp et al. performed a trial in which Australian and Indonesian children aged 6–10 years were supplemented with 88 mg DHA and 22 mg EPA six days a week for 12 months, and the researchers hypothesized that Indonesian children would benefit more from the intervention since they were malnourished [100]. Both experimental groups showed increased plasma DHA and other *n*-3 PUFA levels (although not significant, all *p* > 0.10), but neither group showed improved cognitive assessment battery scores (all *p* > 0.15) [100]. Given the relatively high Australian and Indonesian baseline DHA levels (31.1 ± 10.6 µg/mL and 41.3 ± 13.9 µg/mL, respectively), the effect of DHA + EPA supplementation on cognitive performance in both groups was expected to be minimal [100]. A similar randomized controlled trial performed in Australia supports these results as 12 weeks of 1.6 g of EPA and 1.6 g of DHA daily significantly increased the Omega-3 Index (*p* < 0.001) but did not improve self-regulation nor executive function in 3–5-year-old children (all *p* > 0.18) [99]. In fact, behavioral self-regulation was significantly lower in the intervention group following the intervention (*p* = 0.007) [99]. These trials further support that the supplementation of PUFA-sufficient populations will not improve, and can potentially worsen, cognitive performance regardless of overall nutrition status.

Generally, research in infants shows that early postnatal PUFA intake may not noticeably affect cognitive performance for several months [63]. A randomized controlled trial supplementing 3–9-month-old Gambian infants with 200 mg DHA and 300 mg EPA showed no effect on cognitive development at 9 and 12 months of age [38]. An observational study carried out by Braarud et al. detected a significant positive effect of DHA status at 3 months of age on problem solving at 12 months old of age in Norwegian infants (*p* < 0.05) [58]. Additionally, a sufficiently powered randomized controlled trial conducted by Birch et al. supplemented newborn American infants with ~131 mg DHA or ~131 mg DHA and 270 mg ARA per day from birth to 4 months of age and showed a mean increase of seven points on the Mental Development Index (MDI) of the Bayley Scales of Infant Development assessment at 18 months compared to the control group (*p* < 0.05), and MDI was correlated with plasma and RBC DHA at 4 months of age [106]. Both supplemented groups also had higher developmental age scores on the cognitive and motor subscales of the Bayley Scales at 18 months [106].

Auestad et al. supplemented newborn American infants from birth to 12 months of age with ~51 mg DHA and 165 mg ARA and demonstrated no association between supplementation wand MDI score at 12 months (all *p* > 0.05) [105]. This study may not have detected an association because the DHA and ARA dosages may have been too low, although Auestad et al. supplemented the infants for longer than the previous study and assessed cognition immediately following supplementation. A recent randomized controlled trial supported Auestad et al.’s results by demonstrating that supplementation of 10–16-month (corrected age) preterm infants with 200 mg DHA and 200 mg ARA for six months did not improve cognition as measured by the Bayley Scales of Infant Development-III despite experiencing significant increases in erythrocyte DHA and ARA levels (ARA change: 2.2 mol% (95% CI: 1.7 to 2.8); DHA change: 1.2 mol% (95% CI: 1.0 to 1.5)) [104]. These results collectively suggest that dosage may have a more significant effect than duration and assessment timing, or that the effects of supplementation may not be demonstrated until months after supplementation has seized. More studies should be conducted to elucidate the relationship between PUFA levels and cognition during infancy.

Moreover, researchers have not found an association between maternal or postnatal DHA status and cognitive performance after 2 years of age [51,107]. This is likely because maternal PUFA status greatly influences PUFA status in early infancy, but the effects wean as executive function truly begins to develop, i.e., around 2 years of age [108]. At this point, PUFA sufficiency must be maintained exogenously through the child’s diet, specifically through the consumption of foods high in EPA and DHA, such as fatty fish. Thus, future studies which aim to observe the relationship between early postnatal DHA status and cognition should test cognitive performance between 1 and 2 years of age.

Overall, studies from child populations of varying nutritional status highlighted an association between fatty acid levels and cognitive outcomes. Specifically, sufficient levels of *n*-3 PUFAs EPA and DHA were related to high performance on various cognitive tests. This association was seen in both well-nourished and malnourished populations, which demonstrates that FA deficiency can still be present and result in cognitive impairment regardless of overall nutrition status. The majority of these studies, however, did not examine markers of EFAD, notably LA, ALA, Mead acid, and T:T ratio. So, there is limited research on how EFAD specifically impacts cognitive outcomes and how this relationship may differ among child populations. Thus, researchers should study child FA levels, especially markers of EFAD, and cognitive outcomes in various regions across the globe to ensure accurate identification of affected populations that require assistance through various intervention efforts for the promotion of proper cognitive development into adulthood.

### 4.2. Potential Mechanisms Relating PUFAs and Cognitive Outcomes in Children

Many biochemical mechanisms connect PUFA levels and cognitive function. The foundation for this relationship is PUFAs’ role in phospholipid synthesis. Specifically, PUFAs, notably DHA, are metabolized with uridine and choline to synthesize neuronal membrane phospholipids via the CDP-choline cycle or the Kennedy cycle [109]. A mouse study supported this pathway, as supplementation of ischemic mice’s diets with citicoline (a byproduct of choline) (40 mg/kd/day) and DHA (300 mg/kg daily) for 11 days led to improved learning and memory as well as the prevention of neuronal cell death while slightly increasing levels of phosphatidylcholine, the most abundant brain phospholipid [110]. Recently, the administration of a supplement containing uridine, choline, and DHA from 12 to 48 weeks was shown in two separate clinical trials to improve memory in patients with mild Alzheimer’s disease dementia [111,112].

To further determine a mechanism which links PUFAs to early cognitive development, researchers have studied the negative effects of *n*-3 PUFA deficiency on neurotransmission throughout the first few years of life. A mouse study conducted by Wang et al. demonstrated that ALA deficiency (0.09% of diet) significantly lowered brain DHA levels, increased neuroinflammation and neurodevelopmental delay, and lead to poorer spatial learning and memory compared to ALA sufficiency (2.88% of diet) [113]. These results supplement previous mouse studies which showed that *n*-3 PUFA deficiency can lead to poor behavioral performance through the interruption of neurotransmission [114,115,116]. This observation may begin to explain the cognitive deficits exhibited in human children with chronic *n*-3 PUFA deficiency [34,46,100]. Collectively, these results provide evidence for specific mechanisms through which DHA may promote early cognitive development.

Gene expression pathways are largely responsible for the mechanisms connecting *n*-3 PUFAs to early cognitive development. DHA has been shown to be required for the proper functioning of the transcription factors CREB1 and capsase-3, which regulate the expression of pre- and post-synaptic brain proteins, as well as retinoid receptors such as peroxisome proliferator-activated receptors which regulate synaptic transmission [117,118]. When Chalon et al. replenished the ALA levels in young deficient rats, *n*-3 PUFA levels in the brain increased to standard levels (mean (SD): 30.2 (0.3)% total FA) in brain areas of immense proliferation during early development such as the prefrontal cortex and the hippocampus [119]. An earlier piglet study of ALA replenishment also demonstrated an increase in membrane phospholipids and dopamine levels, which can affect membrane fluidity and consequently brain vesicle formation [120,121]. Despite these positive results, ALA replenishment later than 21 days did not restore dopaminergic pathway activity [119]. Since weaning usually begins around this time, the window of intense cognitive development is likely closed. Collectively, these results support the time-sensitive nature of *n*-3 PUFA deficiency during the early stages of neuronal development, which has the potential to affect cognition later in life.

Overall, various studies support the association between EFA levels and cognitive outcomes in children. Studies which did not observe an association may not have supplemented subjects with a high enough dosage of PUFAs or their study populations exhibited overall EFA sufficiency [100,102,105]. Various animal and human studies have substantiated these associations by connecting PUFAs with cognition and demonstrating the role of PUFAs in cell membrane formation, dopaminergic transmission, and gene expression regulation. Researchers should focus on strengthening these associations by assessing FA levels in LMICs which contain understudied child populations.

## 5. HUFAs, Growth Parameters, and Cognitive Development

Highly unsaturated fatty acids (HUFAs) are the byproducts of EFAs LA and ALA, and the balance of HUFAs determines the overall inflammatory state of the body which has implications for a wide range of health conditions [122]. Specifically, *n*-6 ARA is transformed at a more rapid rate than *n*-3 EPA into downstream prostaglandin byproducts. Thus, an increase in the *n*-6 prostaglandin byproducts prostaglandin D2 and E2 (PGD_2_ and PGE_2_) can lead to an inflammatory state, which can cause cognitive dysfunction, inhibit cell growth, decrease calcium deposition, and reduce bone resorption, which may lead to stunted growth [28,123,124,125,126].

Based on the selective reactivity of HUFAs within the arachidonic acid cascade, relative dietary intake and resulting *n*-3 ALA and *n*-6 LA levels may make certain children more likely to be in an inflammatory state than others. Specifically, individuals in countries with a high-*n*-3 diet may be less likely to develop “harmful cascade outcomes” because high amounts of *n*-3 HUFAs made endogenously or eaten exogenously can outcompete with *n*-6 HUFAs which ultimately results in an anti-neuroinflammatory state [126]. Therefore, these individuals may experience less inflammation and less severe EFAD complications than those with Westernized diets who are more likely to consume a higher amount of LA and other *n*-6 FAs.

Researchers have used mice models to further demonstrate the potential benefit of HUFA balance favoring *n*-3 HUFAs on inflammation and overall health. Pestka et al. demonstrated that in a lupus murine model, those with crystalline silica dust (cSiO_2_) mediated lupus fed a low DHA diet (2 g/day) and a high DHA diet (5 g/day) had significantly higher HUFA scores (the proportion of *n*-3 HUFAs among total HUFAs) in RBCs than those with cSiO_2_ mediated lupus fed a control diet (mean (SEM): 49.29 (4.89)% 73.39 (4.71)% v. 10.83 (1.21)%) [127]. The same group previously observed that an increased HUFA score was associated with decreased autoimmune biomarkers in the lupus murine model [128]. Taken together, these studies provide preliminary evidence for the benefit of increased dietary intake of *n*-3 HUFAs in those who suffer from increased inflammation. More research should observe this effect in individuals with other inflammatory conditions, notably PEM and EFAD, and how this may impact other health outcomes, such as growth and cognition.

Despite the emerging importance of HUFA balance on health, many studies which have looked at the associations between FA levels and developmental outcomes in children have not examined the HUFA score. More recent studies have included measures of the *n*-6:*n*-3 ratio [32,33,36,45,50,59], but this calculation includes FAs which are not highly unsaturated, notably EFAs LA and ALA, which are not directly metabolized to determine an inflammatory state, and excludes the *n*-9 HUFA Mead acid which is used to diagnose EFAD. Thus, this calculation may not as accurately assess potential EFAD and overall health. To specifically look at the relative levels of *n*-3 and *n*-6 HUFAs in an individual, researchers can calculate the HUFA score. The numerator of this calculation represents the sum of all the *n*-3 HUFAs (usually EPA, DPA*n*-3, and DHA), while the denominator is the sum of all HUFAs (*n*-6 HUFAs include DGLA, ARA, DTA, and DPA*n*-6 and *n*-9 HUFAs include Mead acid). The resulting ratio can be multiplied by 100 to yield the *n*-3 HUFA score [122]. Western diets common in HICs and now LMICs may have *n*-3 HUFA scores around 30%, while populations with high fish intake exhibit *n*-3 HUFA scores as high as 70% [128]. Randomized controlled trials looking at the effect of the HUFA score on chronic disease outcomes in U.S. adults demonstrated that a HUFA score of at least 40% resulted in the most significant health improvements [28].

The above values, however, were determined by adult populations. There have not been enough studies measuring the HUFA score in children to determine if these values compare to those in child populations. Even if studies report some HUFA values, such as EPA and DHA, they do not report all values necessary for the proper calculation, which makes it difficult to compare these HUFA scores with studies which measured fatty acid levels more comprehensively. Only one study included in this review measured the HUFA score of the child population [32]. The HUFA score among 6–10-year-old Ugandan children was about 17%, which is even lower than those demonstrated in adult populations with Westernized diets and very far from the optimal HUFA score of 40% [28,32]. This study did not find any associations between HUFA scores and growth, notably HAZ, WAZ, or BAZ scores [32]. No studies measuring the relationship between FA levels and cognitive outcomes in children included in this review reported a HUFA score. Thus, HUFA scores are barely reported among child populations, despite this newfound interest and potential significance of the HUFA score to determine overall health and inflammation which can impact child development.

More evidence is needed to support the importance of the balance of dietary *n*-3 and *n*-6 HUFA intake on the prevention of adverse growth outcomes in children, notably stunting and cognitive function. Optimal HUFA levels (≥40% *n*-3 in HUFA) may prevent neuroinflammation and cell growth irregularities by decreasing the production of PGE2 and PGD2 from the arachidonic cascade. To further validate this theory and take advantage of this underexplored area, researchers should perform more comprehensive blood panels in children which include all the HUFAs necessary to calculate the HUFA score, notably Mead acid and ARA. These two fatty acid levels can then be used to calculate the T:T ratio to determine EFAD in the population. Relating both the HUFA score and the T:T ratio to growth and cognition outcomes in children may provide stronger evidence for the importance of fatty acid sufficiency and the optimization of HUFA levels as an avenue to treat EFAD-related complications in early childhood and prevent adverse developmental outcomes which can have lasting consequences into adulthood.

## 6. Conclusions

In conducting this review, there are some limitations in the current literature regarding the effects of EFAD on growth and cognition. A majority of the cross-sectional and longitudinal studies which monitored fatty acid levels did not measure Mead acid nor calculate the T:T ratio, which is regarded as the gold standard for measuring EFAD [18]. Specifically, this measurement and calculation were not performed for any of the HIC child populations included in this review. This did not allow these studies to measure the association between developmental outcomes and EFAD. EFAD should be screened in all child populations, regardless of country income status, as it is a less apparent form of malnutrition that can potentially lead to growth and cognitive deficits which can last into adulthood. Prioritization, however, should be given to LMIC child populations, as they are historically more at risk for other nutritional deficiencies, notably PEM [2]. PEM may make EFAD worse, as it can lead to the use and degradation of endogenous fatty acids [74,129]. Research in populations with PEM may also be beneficial to further elucidate the relationship between EFAD and growth outcomes, as researchers can control for this factor and reduce its confounding effect.

Many studies included in this review also did not measure associations between HUFA levels and health outcomes, which may be beneficial to support the theory that HUFA levels affect overall health by influencing the inflammatory state of the body [28,122,123,124,125,126]. Although country income status is not necessarily a predictor of the overall fatty acid profile, the lack of resources in LMICs has limited the amount of research describing child population blood fatty acid levels as well as their association with developmental outcomes. From current research, it is apparent that other factors, such as diet, geographical location, and genetics, may play a significant role in determining blood fatty acid levels regardless of country income status. Moving forward, it would be beneficial for researchers to utilize a unified approach to collecting fatty acid values, including the widespread measurement of Mead acid and HUFA levels. In addition, calculating the T:T ratio and HUFA score would make the data not only more reflective of the children’s nutritional status but also aid in the direct comparison between values in a comparable study. Together, these efforts to perform more comprehensive fatty acid analyses in child populations (particularly those in LMICs) should allow researchers and clinicians to develop appropriate interventions to reverse EFAD during sensitive developmental periods to promote proper growth and cognition throughout childhood, adolescence, and adulthood.

## Figures and Tables

**Table 1:** Variation in select blood polyunsaturated fatty acid levels among child populations in different countries and regions.

Country	Study Size	Age	Tissue	LA	DGLA	ARA	DTA	DPA *n*-6	ALA	EPA	DPA *n*-3	DHA	Mead Acid	Study
Burkina Faso	1609	6–23 months	Whole blood	16.2 (2.26)	0.85 (0.21)	7.08 (1.53)	-	0.38 (0.12)	0.21 (0.09)	0.13 (0.10, 0.18) ^1^	0.43 (0.12)	1.64 (0.53)	0.07 (0.03)	[39]
Cambodia	174	4 months–16 years	Whole blood	13.2 (2.7)	0.7 (0.2)	3.6 (1.3)	-	0.2 (0.1)	0.2 (0.1)	0.1 (0.1)	0.2 (0.1)	0.8 (0.3)	0.08 (0.06)	[36]
Canada (Inuit)	167	11–53 months	RBC	15.7 (3.02)	1.9 (0.59)	10.1 (3.37)	1.60 (0.66)	0.30 (0.30)	0.30 (0.14)	0.84 (0.61)	1.54 (0.69)	2.67 (1.52)	-	[37]
China **	94	7–12 years	RBC	14.64 (1.28)	-	16.27 (1.46)	-	-	0.09 (0.07–0.11) ^1^	0.32 (0.26–0.45) ^1^	-	2.75 (0.59)	-	[44]
Colombia	668	5–12 years	Serum	30.3 (3.10)	1.61 (0.38)	5.96 (1.16)	-	-	0.49 (0.15)	0.22 (0.15)	-	2.30 (0.90)	-	[45]
Gambia *	50	3–9 months	Plasma	322 (91.9) ^2^	19.1 (6.49) ^2^	7.25 (1.35) ^2^	-	3.32 (1.24) ^2^	4.76 (2.16) ^2^	1.34 (0.94, 1.72) ^1^	11.9 (3.82) ^2^	4.44 (0.81) ^2^	-	[38]
Ghana (north)	307	2–6 years	Whole blood	20.6 (0.11) ^3^	1.36 (0.01) ^3^	10.8 (0.09) ^3^	1.69 (0.02) ^3^	-	0.18 (0.01) ^3^	0.22 (0.01) ^3^	-	2.62 (0.04) ^3^	0.14 (0.003) ^3^	[46]
Ghana (south)	209	2–6 years	Whole blood	16.7 (1.92)	1.24 (0.23)	9.18 (1.56)	1.03 (0.25)	0.45 (0.13)	0.25 (0.10)	0.80 (0.35)	1.01 (0.21)	5.09 (0.98)	0.09 (0.01)	[47]
India	625	7–13 years	Plasma	27.3 (0.24)	-	9.71 (0.11)	2.80 (0.07)	0.64 (0.02)	-	-	0.18 (0.01)	0.98 (0.03)	-	[48]
Italy	728	2–9 years	Whole blood	19.4 (2.73)	1.41 (0.15)	9.01 (0.82)	1.06 (0.06)	0.26 (0.05)	0.24 (0.07)	0.69 (0.46)	0.96 (0.20)	2.69 (0.66)	-	[49]
Nepal	303	0–12 months	RBC	11.3	-	13.2	-	-	0.2	0.3	1.6	4.9	-	[50]
Netherlands	306	7–8 years	Plasma	23.2 (2.3)	-	9.2 (1.2)	-	0.32 (0.08)	0.19 (0.07)	0.51 (0.22)	-	2.8 (0.7)	-	[51]
Pakistan **	26	0–5 years	RBC	8.83 (5.67–13.62) ^4^	1.60 (1.25–2.36) ^4^	14.68	3.05 (2.13–3.85) ^4^	1.08 (0.78–2.16) ^4^	0.17 (0.07–0.35) ^4^	0.24 (0.13–0.94) ^4^	1.40 (0.62–2.91) ^4^	2.90 (1.72–4.04) ^4^	0.37 (0.14–1.32) ^4^	[31]
South Africa **	183	7–9 years	Plasma PC	-	-	10.4 (1.67)	-	-	-	0.70 (0.41)	-	4.53 (1.25)	-	[52]
South Korea **	218	4–6 years	RBC	13.6 (1.3)	-	15.3 (1.6)	3.0 (0.6)	-	-	0.8 (0.3)	1.6 (0.3)	8.3 (1.3)	-	[35]
Tanzania	238	3 months	Plasma	20.73 (2.77)	1.95 (0.50)	8.35 (1.83)	0.35 (0.10)	-	0.32 (0.15)	0.37 (0.19)	0.46 (0.13)	2.96 (0.90)	-	[53]
Tanzania	130	4–6 years	Whole Blood	17.58 (2.74)	1.77 (0.42)	10.0 (1.65)	1.35 (0.32)	0.78 (0.16)	0.41 (0.19)	0.43 (0.18)	0.95 (0.26)	2.94 (0.76)	0.14 (0.06)	[54]
Uganda	240	6–10 years	Serum	36.8 (0.35) ^3^	0.56 (0.02) ^3^	11.8 (0.15) ^3^	0.018 (0.001) ^3^	0.014 (0.001) ^3^	0.26 (0.01) ^3^	0.46 (0.02) ^3^	0.067 (0.003) ^3^	2.11 (0.06) ^3^	0.085 (0.004) ^3^	[32,33]
United Kingdom	493	7–9 years	Whole blood	19.2 (2.29)	1.56 (0.34)	8.17 (1.31)	-	0.25 (0.10)	0.54 (0.26)	0.56 (0.20)	1.03 (0.27)	1.9 (0.53)	-	[34]

All FA values are % total mean (SD) reported unless specified otherwise; * All values presented for this study are endpoints for the control group; ** All values presented for this study are baseline for control group; ^1^ Values expressed as % total median (25th, 75th percentile); ^2^ Values expressed as ng /µL mean (SD); ^3^ Values expressed as % total mean (SE); ^4^ Values expressed as % total median (range); LA = linoleic acid; DGLA = dihomo-gamma-linolenic acid; ARA = arachidonic acid; DTA = docosatetraenoic acid; DPA = docosapentaenoic acid; ALA = α-linolenic acid; EPA = eicosapentaenoic acid; DHA = docosahexaenoic acid; RBC = red blood cell; PC = phosphatidylcholine.

**Table 3:** Associations between blood fatty acid levels and cognitive outcomes among child populations in different countries and regions.

Country	Study Size	Age	Design	Intervention	Tissue	Cognitive Outcome Measured	Fatty Acids Measured	Main Outcomes	Study
Australia	78	3–5 years	RCT	1.3 g DHA + 0.3 g EPA/day via powder in milk/yogurt for 12 weeks	RBC	Head, Toes, Knees Shoulders (HTKS) task and Child-Self-Regulation and Social Behavior Questionnaire (CSBQ), Go/No-Go and Mr. Ant tasks, Behavior Rating Inventory of Executive Function- Preschool Version (BRIEF-P)	Omega-3 Index	DHA + EPA supplementation significantly increased Omega-3 Index Post-intervention CSBQ behavioral self-regulation was significantly higher in the placebo group Supplementation did not improve self-regulation or executive function outcomes	[99]
Australia	396	6–10 years	RCT	88 mg DHA and 22 mg EPA in powder form 6 days a week for 12 months	Plasma	Weschler Intelligence Scale for Children III (WISC-III), Developmental Neuropsychological Assessment (NEPSY), Rey Auditory Verbal Learning Test (RAVLT)	ALA, EPA, DPA, DHA, total *n*-3 PUFAs	No effect of DHA + EPA supplementation on cognitive test performance	[100]
China	94	7–12 years	RCT	800 mg DHA/day via capsule for 6 months	RBC	Digit Span Backwards and Wisconsin card sorting test at 3 and 6 months	SFAs, MUFAs, *n*-3 and *n*-6 PUFAs, *n*-6:*n*-3 ratio	Both the intervention and control groups showed a significant increase in Digit Span Backwards and Wisconsin card sorting test scores No effect of DHA supplementation on test scores	[44]
Gambia	50	3–9 months	RCT	200 mg DHA and 300 mg EPA or 2 mL olive oil/day for 6 months	Plasma	Willatts Infant Planning Test	*n*-3 and *n*-6 PUFAs	PUFA supplementation did not impact cognitive performance at 9 nor 12 months of age	[38]
Ghana (north)	307	2–6 years	Cross sectional	-	Whole blood	Dimensional change card sort (DCCS) total pass	SFA, MUFA, *n*-3, *n*-6, and *n*-9 PUFAs, T:T ratio, ARA:EPA	FA levels were associated with variation in DCCS performance Positive association between DCCS total pass and DHA and ALA levels Children with highest levels of DHA, DHA + EPA, and total *n*-3 PUFAs were more likely to pass at least one condition of DCCS test	[46]
Indonesia	384	6–10 years	RCT	88 mg DHA and 22 mg EPA in powder form 6 days a week for 12 months	Plasma	WISC-III, NEPSY, RAVLT	ALA, EPA, DPA, DHA, total *n*-3 PUFAs	No effect of DHA + EPA supplementation on cognitive test performance	[100]
Malawi	2565	6–59 months	RCT	DHA + high-oleic (HO) ready-to-use therapeutic food (RUTF) or HO RUTF for 1 month	Plasma PL	Malawi Developmental Assessment Tool (MDAT) global z-score and Willatts problem-solving assessment (PSA) intention score	*n*-3 and *n*-6 PUFAs	DHA + HO RUTF and HO RUTF supplementation increased plasma PL EPA and ALA levels DHA + HO RUTF also increased plasma PL DHA levels Children with severe acute malnutrition who received DHA + HO RUTF had higher gross motor and social domain MDAT scores than those who received standard RUTF PSA scores did not differ between study groups	[101]
Netherlands	304	7 years	Longitudinal	-	Plasma PL	Kaufman Assessment Battery for Children	*n*-3 and *n*-6 PUFAs	PUFA levels at birth and 7 years were not associated with scores from the Kaufman Assessment Battery for Children at 7 years old	[51]
Norway	82	1 year	Longitudinal	-	RBC	Ages and Stages Questionnaire	SFA, MUFA, *n*-3 and *n*-6 PUFAs, Omega-3 Index	DHA status at three months was positively associated with problem solving at 12 months	[58]
Norway	218	4–6 years	RCT	~1000 mg EPA + DHA from fatty fish or red meat for 4 months	RBC	Wechsler Preschool and Primary Scale of Intelligence III (WPPSI-III) and 9-Hole Peg Test	SFA, MUFA, *n*-3 and *n*-6 PUFAs, Omega-3 Index	Intent-to-treat analysis did not show an effect of fatty fish supplementation on cognitive outcomes After adjusting for dietary compliance, fatty fish supplementation showed a higher improvement in WPPSI-III total raw score, vocabulary score, block design score, and symbol search score	[102]
Oman	132	9–10 years	RCT	403 mg DHA/weekday from fish oil capsule or 150–200 DHA mg/weekday from grilled fish for 12 weeks	RBC	Verbal Fluency Test, Buschke Selective Reminding Test, Trail Making Test	EPA, DHA, DPA	Both fish oil and grilled fish supplementation showed a positive effect on Trail Making Test Part B score, but capsule supplementation showed a significantly greater positive effect	[103]
South Africa	183	7–9 years	RCT	192 mg DHA and 82 mg EPA/weekday in fish spread for 6 months (104 days)	Plasma and RBC PC	Hopkins Verbal Learning Test (HVLT), Afrikaans reading test, Afrikaans spelling test	EPA, DHA, ARA	Fish spread supplementation yielded positive intervention effects for HVLT recognition index, HVLT discrimination index, and spelling test score After adjusting for dietary compliance, fish spread supplementation yielded a positive intervention effect for above measures and reading test score	[52]
Tanzania	130	4–6 years	Cross sectional	-	Whole blood	DCCS total pass and highest test passed	SFA, *n*-3, *n*-6, and *n*-9 PUFAs, T:T ratio	FA levels were associated with variation in DCCS performance LA levels were positively associated with DCCS performance, and ALA levels were negatively associated with DCCS performance Absence of EFAD levels is associated with increased likelihood of successful DCCS performance	[54]
United Kingdom	493	7–9 years	Cross sectional	-	Whole blood	British Ability Scales II Word Reading Achievement subtest and Recall of Digits Forward and Recall of Digits Backward sub-tests	*n*-3 and *n*-6 PUFAs	Reading scores were positively associated with DHA, EPA, DPA, DHA+EPA, and total *n*-6 PUFAs Working memory was positively associated with DHA, EPA, DPA, DHA+EPA, and total *n*-3 PUFAs	[34]
United States	338	10–16 months	RCT	200 mg DHA + 200 mg ARA/day in powder form in milk for 6 months	RBC	Bayley Scales of Infant Development III (BSID-III) cognitive composite score	*n*-3 and *n*-6 PUFAs, *n*-6:*n*-3 ratio	RBC DHA and ARA levels significantly increased with supplementation Mean baseline BSID-III cognitive composite scores did not improve with DHA and ARA supplementation	[104]
United States	239	12 months	RCT	~51 mg DHA + ~165 mg ARA in fish/fungal oil or egg-derived triglyceride-supplemented formula for 12 months	RBC PC, RBC PE	Fagan Test of Infant Intelligence (6 and 9 months), BSID-II (6 and 12 months), MacArthur Communicative Development Inventories (9 and 14 months)	ARA, DHA	Neither supplementation affected cognitive test performance at any age	[105]
United States	56	18 months	RCT	~131 mg DHA or ~131 mg DHA + 270 mg ARA/can of formula for 16 weeks after birth	Plasma RBC	BSID-II	LA, ALA, ARA, EPA, DHA	DHA + ARA supplementation yielded an average 7-point Mental Development Index (MDI) increase, and DHA supplementation yielded an average 4-point MDI increase at 18 months Both DHA + ARA and DHA-supplemented children had higher developmental age scores on the cognitive and motor subscales of BSID-II at 18 months Plasma and RBC DHA at 4 months of age were correlated with MDI at 18 months of age	[106]

RCT = randomized controlled trial; RBC = red blood cells; PL = phospholipid; PC = phosphatidylcholine; PE = phosphatidylethanolamine; SFA = saturated fatty acid, MUFA = monounsaturated fatty acid, PUFA = polyunsaturated fatty acid; LA = linoleic acid; ALA = α-linolenic acid; ARA = arachidonic acid; EPA = eicosapentaenoic acid; DHA = docosahexaenoic acid.

## Data Availability

Not applicable.

## References

[1] World Health Organization Malnutrition. https://www.who.int/news-room/fact-sheets/detail/malnutrition.

[2] World Health Organization (2021). Levels and Trends in Child Malnutrition: UNICEF/WHO/The World Bank Group Joint Child Malnutrition Estimates: Key Findings of the 2021 Edition.

[3] de Onis M., Branca F. (2016). Childhood stunting: A global perspective. Matern. Child Nutr..

[4] Suryawan A., Jalaludin M.Y., Poh B.K., Sanusi R., Tan V.M.H., Geurts J.M., Muhardi L. (2022). Malnutrition in early life and its neurodevelopmental and cognitive consequences: A scoping review. Nutr. Res. Rev..

[5] Christian P., Smith E.R. (2018). Adolescent Undernutrition: Global Burden, Physiology, and Nutritional Risks. Ann. Nutr. Metab..

[6] Jeyaseelan V., Jeyaseelan L., Yadav B. (2016). Incidence of, and risk factors for, malnutrition among children aged 5–7 years in South India. J. Biosoc. Sci..

[7] Obasohan P.E., Walters S.J., Jacques R., Khatab K. (2020). Risk Factors Associated with Malnutrition among Children Under-Five Years in Sub-Saharan African Countries: A Scoping Review. Int. J. Environ. Res. Public Health.

[8] Bernard J.Y., Pan H., Aris I.M., Moreno-Betancur M., Soh S.E., Yap F., Tan K.H., Shek L.P., Chong Y.S., Gluckman P.D. (2018). Long-chain polyunsaturated fatty acids, gestation duration, and birth size: A Mendelian randomization study using fatty acid desaturase variants. Am. J. Clin. Nutr..

[9] Adetunji H., Salami G., Fadil M., Zaman T., Bakri M., Khalil M., Bushara M. (2019). Relative Merits of Selected Anthropometric Measurements for Detecting Protein-Energy-Malnutrition (PEM) in Children Under Five Years in a Resource Limited Setting. Int. J. Med. Public Health.

[10] Teigen L.M., Kuchnia A.J., Nagel E.M., Price K.L., Hurt R.T., Earthman C.P. (2018). Diagnosing clinical malnutrition: Perspectives from the past and implications for the future. Clin. Nutr. ESPEN.

[11] Isabirye N., Bukenya J.N., Nakafeero M., Ssekamatte T., Guwatudde D., Fawzi W. (2020). Dietary diversity and associated factors among adolescents in eastern Uganda: A cross-sectional study. BMC Public Health.

[12] Kabwama S.N., Bahendeka S.K., Wesonga R., Mutungi G., Guwatudde D. (2019). Low consumption of fruits and vegetables among adults in Uganda: Findings from a countrywide cross-sectional survey. Arch. Public Health.

[13] Murray C.J.L., Aravkin A.Y., Zheng P., Abbafati C., Abbas K.M., Abbasi-Kangevari M., Abd-Allah F., Abdelalim A., Abdollahi M., Abdollahpour I. (2020). Global burden of 87 risk factors in 204 countries and territories, 1990–2019: A systematic analysis for the Global Burden of Disease Study 2019. Lancet.

[14] Rincón-Cervera M., Valenzuela R., Hernandez-Rodas M.C., Barrera C., Espinosa A., Marambio M., Valenzuela A. (2016). Vegetable oils rich in alpha linolenic acid increment hepatic n-3 LCPUFA, modulating the fatty acid metabolism and antioxidant response in rats. Prostaglandins Leukot Essent Fat. Acids.

[15] Cormier H., Rudkowska I., Lemieux S., Couture P., Julien P., Vohl M.C. (2014). Effects of FADS and ELOVL polymorphisms on indexes of desaturase and elongase activities: Results from a pre-post fish oil supplementation. Genes Nutr..

[16] Czumaj A., Śledziński T. (2020). Biological Role of Unsaturated Fatty Acid Desaturases in Health and Disease. Nutrients.

[17] Gramlich L., Ireton-Jones C., Miles J.M., Morrison M., Pontes-Arruda A. (2019). Essential Fatty Acid Requirements and Intravenous Lipid Emulsions. JPEN J. Parenter. Enteral Nutr..

[18] Siguel E.N., Chee K.M., Gong J.X., Schaefer E.J. (1987). Criteria for essential fatty acid deficiency in plasma as assessed by capillary column gas-liquid chromatography. Clin. Chem..

[19] Holman R.T., Johnson S.B., Mercuri O., Itarte H.J., Rodrigo M.A., De Tomas M.E. (1981). Essential fatty acid deficiency in malnourished children. Am. J. Clin. Nutr..

[20] Anez-Bustillos L., Dao D.T., Fell G.L., Baker M.A., Gura K.M., Bistrian B.R., Puder M. (2018). Redefining essential fatty acids in the era of novel intravenous lipid emulsions. Clin. Nutr..

[21] Carey A.N., Rudie C., Mitchell P.D., Raphael B.P., Gura K.M., Puder M. (2019). Essential Fatty Acid Status in Surgical Infants Receiving Parenteral Nutrition With a Composite Lipid Emulsion: A Case Series. JPEN J. Parenter. Enteral Nutr..

[22] Memon N., Hussein K., Hegyi T., Herdt A., Griffin I.J. (2019). Essential Fatty Acid Deficiency with SMOFlipid Reduction in an Infant with Intestinal Failure-Associated Liver Disease. JPEN J. Parenter. Enteral Nutr..

[23] Mirza F.N., Leventhal J.S. (2020). The rash in life-threatening metabolic and endocrine disorders. Clin. Dermatol..

[24] Mariamenatu A.H., Abdu E.M. (2021). Overconsumption of Omega-6 Polyunsaturated Fatty Acids (PUFAs) versus Deficiency of Omega-3 PUFAs in Modern-Day Diets: The Disturbing Factor for Their “Balanced Antagonistic Metabolic Functions” in the Human Body. J. Lipids.

[25] Deoni S., Dean D., Joelson S., O’Regan J., Schneider N. (2018). Early nutrition influences developmental myelination and cognition in infants and young children. Neuroimage.

[26] Van Dael P. (2021). Role of n-3 long-chain polyunsaturated fatty acids in human nutrition and health: Review of recent studies and recommendations. Nutr. Res. Pract..

[27] Willatts P. (2018). Effects of Nutrition on the Development of Higher-Order Cognition. Nestlé Nutr. Inst. Workshop Ser..

[28] Lands B. (2015). Omega-3 PUFAs Lower the Propensity for Arachidonic Acid Cascade Overreactions. Biomed. Res. Int..

[29] Brew B.K., Toelle B.G., Webb K.L., Almqvist C., Marks G.B. (2015). Omega-3 supplementation during the first 5 years of life and later academic performance: A randomised controlled trial. Eur. J. Clin. Nutr..

[30] Teisen M.N., Vuholm S., Niclasen J., Aristizabal-Henao J.J., Stark K.D., Geertsen S.S., Damsgaard C.T., Lauritzen L. (2020). Effects of oily fish intake on cognitive and socioemotional function in healthy 8-9-year-old children: The FiSK Junior randomized trial. Am. J. Clin. Nutr..

[31] Smit E.N., Dijkstra J.M., Schnater T.A., Seerat E., Muskiet F.A., Boersma E.R. (1997). Effects of malnutrition on the erythrocyte fatty acid composition and plasma vitamin E levels of Pakistani children. Acta Paediatr..

[32] Jain R., Ezeamama A.E., Sikorskii A., Yakah W., Zalwango S., Musoke P., Boivin M.J., Fenton J.I. (2019). Serum n-6 Fatty Acids are Positively Associated with Growth in 6-to-10-Year Old Ugandan Children Regardless of HIV Status-A Cross-Sectional Study. Nutrients.

[33] Pobee R.A., Fenton J.I., Sikorskii A., Zalwango S.K., Felzer-Kim I., Medina I.M., Giordani B., Ezeamama A.E. (2022). Association of serum PUFA and linear growth over 12 months among 6-10 years old Ugandan children with or without HIV. Public Health Nutr..

[34] Montgomery P., Burton J.R., Sewell R.P., Spreckelsen T.F., Richardson A.J. (2013). Low blood long chain omega-3 fatty acids in UK children are associated with poor cognitive performance and behavior: A cross-sectional analysis from the DOLAB study. PLoS ONE.

[35] Hwang I., Cha A., Lee H., Yoon H., Yoon T., Cho B., Lee S., Park Y. (2007). N-3 polyunsaturated fatty acids and atopy in Korean preschoolers. Lipids.

[36] Sigh S., Lauritzen L., Wieringa F.T., Laillou A., Chamnan C., Angkeabos N., Moniboth D., Berger J., Stark K.D., Roos N. (2020). Whole-blood PUFA and associations with markers of nutritional and health status in acutely malnourished children in Cambodia. Public Health Nutr..

[37] Blanchet R., Lauziere J., Gagne D., Vezina C., Ayotte P., O’Brien H.T. (2014). Usual dietary fatty acid intakes and red-blood-cell membrane fatty acid composition in Inuit children attending child-care centres in Nunavik, northern Quebec, Canada. Public Health Nutr..

[38] van der Merwe L.F., Moore S.E., Fulford A.J., Halliday K.E., Drammeh S., Young S., Prentice A.M. (2013). Long-chain PUFA supplementation in rural African infants: A randomized controlled trial of effects on gut integrity, growth, and cognitive development. Am. J. Clin. Nutr..

[39] Yaméogo C.W., Cichon B., Fabiansen C., Rytter M.J.H., Faurholt-Jepsen D., Stark K.D., Briend A., Shepherd S., Traoré A.S., Christensen V.B. (2017). Correlates of whole-blood polyunsaturated fatty acids among young children with moderate acute malnutrition. Nutr. J..

[40] Gurzell E.A., Wiesinger J.A., Morkam C., Hemmrich S., Harris W.S., Fenton J.I. (2014). Is the omega-3 index a valid marker of intestinal membrane phospholipid EPA+DHA content?. Prostaglandins Leukot Essent Fat. Acids.

[41] Harris W.S., Varvel S.A., Pottala J.V., Warnick G.R., McConnell J.P. (2013). Comparative effects of an acute dose of fish oil on omega-3 fatty acid levels in red blood cells versus plasma: Implications for clinical utility. J. Clin. Lipidol..

[42] Micalizzi G., Ragosta E., Farnetti S., Dugo P., Tranchida P.Q., Mondello L., Rigano F. (2020). Rapid and miniaturized qualitative and quantitative gas chromatography profiling of human blood total fatty acids. Anal. Bioanal. Chem..

[43] Fenton J.I., Gurzell E.A., Davidson E.A., Harris W.S. (2016). Red blood cell PUFAs reflect the phospholipid PUFA composition of major organs. Prostaglandins Leukot Essent Fat. Acids.

[44] Yang G.Y., Wu T., Huang S.Y., Huang B.X., Wang H.L., Lan Q.Y., Li C.L., Zhu H.L., Fang A.P. (2021). No effect of 6-month supplementation with 300 mg/d docosahexaenoic acid on executive functions among healthy school-aged children: A randomized, double-blind, placebo-controlled trial. Eur. J. Nutr..

[45] Perng W., Villamor E., Mora-Plazas M., Marin C., Baylin A. (2015). Alpha-linolenic acid (ALA) is inversely related to development of adiposity in school-age children. Eur. J. Clin. Nutr..

[46] Adjepong M., Yakah W., Harris W.S., Annan R.A., Pontifex M.B., Fenton J.I. (2018). Whole blood n-3 fatty acids are associated with executive function in 2-6-year-old Northern Ghanaian children. J. Nutr. Biochem..

[47] Adjepong M., Yakah W., Harris W.S., Colecraft E., Marquis G.S., Fenton J.I. (2018). Association of Whole Blood Fatty Acids and Growth in Southern Ghanaian Children 2(-)6 Years of Age. Nutrients.

[48] Parasannanavar D., Gaddam I., Bukya T., Ibrahim S.A., Reddy K.S., Banjara S.K., Salvadi B.P.P., Kumar B.N., Rao S.F., Geddam J.J.B. (2021). Omega-3 polyunsaturated fatty acid intake and plasma fatty acids of school going Indian children—A cross-sectional study. Prostaglandins Leukot Essent Fat. Acids.

[49] Wolters M., Schlenz H., Bornhorst C., Rise P., Galli C., Moreno L.A., Pala V., Siani A., Veidebaum T., Tornaritis M. (2015). Desaturase Activity Is Associated With Weight Status and Metabolic Risk Markers in Young Children. J. Clin. Endocrinol. Metab..

[50] Henjum S., Lie Ø., Ulak M., Thorne-Lyman A.L., Chandyo R.K., Shrestha P.S., Wafaie W.F., Strand T.A., Kjellevold M. (2018). Erythrocyte fatty acid composition of Nepal breast-fed infants. Eur. J. Nutr..

[51] Bakker E.C., Ghys A.J., Kester A.D., Vles J.S., Dubas J.S., Blanco C.E., Hornstra G. (2003). Long-chain polyunsaturated fatty acids at birth and cognitive function at 7 y of age. Eur. J. Clin. Nutr..

[52] Dalton A., Wolmarans P., Witthuhn R.C., van Stuijvenberg M.E., Swanevelder S.A., Smuts C.M. (2009). A randomised control trial in schoolchildren showed improvement in cognitive function after consuming a bread spread, containing fish flour from a marine source. Prostaglandins Leukot Essent Fat. Acids.

[53] Kamenju P., Hertzmark E., Kabagambe E.K., Smith E.R., Muhihi A., Noor R.A., Mshamu S., Briegleb C., Sudfeld C., Masanja H. (2020). Factors associated with plasma n-3 and n-6 polyunsaturated fatty acid levels in Tanzanian infants. Eur. J. Clin. Nutr..

[54] Jumbe T., Comstock S.S., Harris W.S., Kinabo J., Pontifex M.B., Fenton J.I. (2016). Whole-blood fatty acids are associated with executive function in Tanzanian children aged 4-6 years: A cross-sectional study. Br. J. Nutr..

[55] Forsyth S., Gautier S., Salem N. (2016). Global Estimates of Dietary Intake of Docosahexaenoic Acid and Arachidonic Acid in Developing and Developed Countries. Ann. Nutr. Metab..

[56] Hadley K.B., Ryan A.S., Forsyth S., Gautier S., Salem N. (2016). The Essentiality of Arachidonic Acid in Infant Development. Nutrients.

[57] Micha R., Khatibzadeh S., Shi P., Fahimi S., Lim S., Andrews K.G., Engell R.E., Powles J., Ezzati M., Mozaffarian D. (2014). Global, regional, and national consumption levels of dietary fats and oils in 1990 and 2010: A systematic analysis including 266 country-specific nutrition surveys. BMJ.

[58] Braarud H.C., Markhus M.W., Skotheim S., Stormark K.M., Froyland L., Graff I.E., Kjellevold M. (2018). Maternal DHA Status during Pregnancy Has a Positive Impact on Infant Problem Solving: A Norwegian Prospective Observation Study. Nutrients.

[59] Adjepong M., Pickens C.A., Jain R., Harris W.S., Annan R.A., Fenton J.I. (2018). Association of whole blood n-6 fatty acids with stunting in 2-to-6-year-old Northern Ghanaian children: A cross-sectional study. PLoS ONE.

[60] Tosi F., Sartori F., Guarini P., Olivieri O., Martinelli N. (2014). Delta-5 and delta-6 desaturases: Crucial enzymes in polyunsaturated fatty acid-related pathways with pleiotropic influences in health and disease. Adv. Exp. Med. Biol..

[61] Fumagalli M., Moltke I., Grarup N., Racimo F., Bjerregaard P., Jorgensen M.E., Korneliussen T.S., Gerbault P., Skotte L., Linneberg A. (2015). Greenlandic Inuit show genetic signatures of diet and climate adaptation. Science.

[62] Brayner B., Kaur G., Keske M.A., Livingstone K.M. (2018). FADS Polymorphism, Omega-3 Fatty Acids and Diabetes Risk: A Systematic Review. Nutrients.

[63] Carlson S.J., Fallon E.M., Kalish B.T., Gura K.M., Puder M. (2013). The role of the ω-3 fatty acid DHA in the human life cycle. JPEN J. Parenter. Enteral Nutr..

[64] Darcey V.L., McQuaid G.A., Fishbein D.H., VanMeter J.W. (2020). Relationship between whole blood omega-3 fatty acid levels and dorsal cingulate gray matter volume: Sex differences and implications for impulse control. Nutr. Neurosci..

[65] Falomir-Lockhart L.J., Cavazzutti G.F., Giménez E., Toscani A.M. (2019). Fatty Acid Signaling Mechanisms in Neural Cells: Fatty Acid Receptors. Front. Cell Neurosci..

[66] Semba R.D., Trehan I., Li X., Salem N., Moaddel R., Ordiz M.I., Maleta K.M., Kraemer K., Manary M.J. (2017). Low serum ω-3 and ω-6 polyunsaturated fatty acids and other metabolites are associated with poor linear growth in young children from rural Malawi. Am. J. Clin. Nutr..

[67] Harris C., Demmelmair H., von Berg A., Lehmann I., Flexeder C., Koletzko B., Heinrich J., Standl M. (2017). Associations between fatty acids and low-grade inflammation in children from the LISAplus birth cohort study. Eur. J. Clin. Nutr..

[68] Jumbe T., Comstock S.S., Hahn S.L., Harris W.S., Kinabo J., Fenton J.I. (2016). Whole Blood Levels of the n-6 Essential Fatty Acid Linoleic Acid Are Inversely Associated with Stunting in 2-to-6 Year Old Tanzanian Children: A Cross-Sectional Study. PLoS ONE.

[69] Stratakis N., Gielen M., Margetaki K., de Groot R.H.M., Apostolaki M., Chalkiadaki G., Vafeiadi M., Leventakou V., Karachaliou M., Godschalk R.W. (2019). Polyunsaturated fatty acid status at birth, childhood growth, and cardiometabolic risk: A pooled analysis of the MEFAB and RHEA cohorts. Eur. J. Clin. Nutr..

[70] Muthayya S., Eilander A., Transler C., Thomas T., van der Knaap H.C., Srinivasan K., van Klinken B.J., Osendarp S.J., Kurpad A.V. (2009). Effect of fortification with multiple micronutrients and n-3 fatty acids on growth and cognitive performance in Indian schoolchildren: The CHAMPION (Children’s Health and Mental Performance Influenced by Optimal Nutrition) Study. Am. J. Clin. Nutr..

[71] Rivera-Pasquel M., Flores-Aldana M., Parra-Cabrera M.S., Quezada-Sánchez A.D., García-Guerra A., Maldonado-Hernández J. (2020). Effect of Milk-Based Infant Formula Fortified with PUFAs on Lipid Profile, Growth and Micronutrient Status of Young Children: A Randomized Double-Blind Clinical Trial. Nutrients.

[72] O’Tierney-Ginn P.F., Davina D., Gillingham M., Barker D.J.P., Morris C., Thornburg K.L. (2017). Neonatal fatty acid profiles are correlated with infant growth measures at 6 months. J. Dev. Orig. Health Dis..

[73] Ingol T.T., Li R., Boone K.M., Rausch J., Klebanoff M.A., Turner A.N., Yeates K.O., Nelin M.A., Sheppard K.W., Keim S.A. (2019). Docosahexaenoic and Arachidonic Acid Supplementation of Toddlers Born Preterm Does Not Affect Short-Term Growth or Adiposity. J. Nutr..

[74] Schönfeldt H.C., Gibson Hall N. (2012). Dietary protein quality and malnutrition in Africa. Br. J. Nutr..

[75] Hoile S.P., Clarke-Harris R., Huang R.C., Calder P.C., Mori T.A., Beilin L.J., Lillycrop K.A., Burdge G.C. (2014). Supplementation with N-3 long-chain polyunsaturated fatty acids or olive oil in men and women with renal disease induces differential changes in the DNA methylation of FADS2 and ELOVL5 in peripheral blood mononuclear cells. PLoS ONE.

[76] Zuccarello D., Sorrentino U., Brasson V., Marin L., Piccolo C., Capalbo A., Andrisani A., Cassina M. (2022). Epigenetics of pregnancy: Looking beyond the DNA code. J. Assist. Reprod. Genet..

[77] Howard T.D., Mathias R.A., Seeds M.C., Herrington D.M., Hixson J.E., Shimmin L.C., Hawkins G.A., Sellers M., Ainsworth H.C., Sergeant S. (2014). DNA methylation in an enhancer region of the FADS cluster is associated with FADS activity in human liver. PLoS ONE.

[78] Rancourt R.C., Harris H.R., Barault L., Michels K.B. (2013). The prevalence of loss of imprinting of H19 and IGF2 at birth. Faseb J..

[79] Lee H.-S., Barraza-Villarreal A., Biessy C., Duarte-Salles T., Sly P.D., Ramakrishnan U., Rivera J., Herceg Z., Romieu I. (2014). Dietary supplementation with polyunsaturated fatty acid during pregnancy modulates DNA methylation at IGF2/H19 imprinted genes and growth of infants. Physiol. Genom..

[80] Worsch S., Heikenwalder M., Hauner H., Bader B.L. (2018). Dietary n-3 long-chain polyunsaturated fatty acids upregulate energy dissipating metabolic pathways conveying anti-obesogenic effects in mice. Nutr. Metab..

[81] Echeverría F., Valenzuela R., Bustamante A., Álvarez D., Ortiz M., Espinosa A., Illesca P., Gonzalez-Mañan D., Videla L.A. (2019). High-fat diet induces mouse liver steatosis with a concomitant decline in energy metabolism: Attenuation by eicosapentaenoic acid (EPA) or hydroxytyrosol (HT) supplementation and the additive effects upon EPA and HT co-administration. Food Funct..

[82] Kasbi-Chadli F., Ferchaud-Roucher V., Krempf M., Ouguerram K. (2016). Direct and maternal n-3 long-chain polyunsaturated fatty acid supplementation improved triglyceridemia and glycemia through the regulation of hepatic and muscle sphingolipid synthesis in offspring hamsters fed a high-fat diet. Eur. J. Nutr..

[83] Yuan Q., Xie F., Huang W., Hu M., Yan Q., Chen Z., Zheng Y., Liu L. (2022). The review of alpha-linolenic acid: Sources, metabolism, and pharmacology. Phytother. Res..

[84] Kim Y., Kelly O.J., Ilich J.Z. (2013). Synergism of α-linolenic acid, conjugated linoleic acid and calcium in decreasing adipocyte and increasing osteoblast cell growth. Lipids.

[85] Fang X.L., Shu G., Zhang Z.Q., Wang S.B., Zhu X.T., Gao P., Xi Q.Y., Zhang Y.L., Jiang Q.Y. (2012). Roles of α-linolenic acid on IGF-I secretion and GH/IGF system gene expression in porcine primary hepatocytes. Mol. Biol. Rep..

[86] Abshirini M., Ilesanmi-Oyelere B.L., Kruger M.C. (2021). Potential modulatory mechanisms of action by long-chain polyunsaturated fatty acids on bone cell and chondrocyte metabolism. Prog. Lipid Res..

[87] Li Q., Brendemuhl J.H., Jeong K.C., Badinga L. (2014). Effects of dietary omega-3 polyunsaturated fatty acids on growth and immune response of weanling pigs. J. Anim. Sci. Technol..

[88] Bourdon C., Lelijveld N., Thompson D., Dalvi P.S., Gonzales G.B., Wang D., Alipour M., Wine E., Chimwezi E., Wells J.C. (2019). Metabolomics in plasma of Malawian children 7 years after surviving severe acute malnutrition: “ChroSAM” a cohort study. EBioMedicine.

[89] Bonnet N., Ferrari S.L. (2011). Effects of long-term supplementation with omega-3 fatty acids on longitudinal changes in bone mass and microstructure in mice. J. Nutr. Biochem..

[90] Young L.R., Kurzer M.S., Thomas W., Redmon J.B., Raatz S.K. (2013). Low-fat diet with omega-3 fatty acids increases plasma insulin-like growth factor concentration in healthy postmenopausal women. Nutr. Res..

[91] Gholamhosseini S., Nematipour E., Djazayery A., Javanbakht M.H., Koohdani F., Zareei M., Djalali M. (2015). ω-3 fatty acid differentially modulated serum levels of IGF1 and IGFBP3 in men with CVD: A randomized, double-blind placebo-controlled study. Nutrition.

[92] Henriksen C., Almaas A.N., Westerberg A.C., Drevon C.A., Iversen P.O., Nakstad B. (2016). Growth, metabolic markers, and cognition in 8-year old children born prematurely, follow-up of a randomized controlled trial with essential fatty acids. Eur. J. Pediatr..

[93] McNamara R.K., Asch R.H., Lindquist D.M., Krikorian R. (2018). Role of polyunsaturated fatty acids in human brain structure and function across the lifespan: An update on neuroimaging findings. Prostaglandins Leukot Essent Fat. Acids.

[94] Macaron T., Giudici K.V., Bowman G.L., Sinclair A., Stephan E., Vellas B., de Souto Barreto P. (2021). Associations of Omega-3 fatty acids with brain morphology and volume in cognitively healthy older adults: A narrative review. Ageing Res. Rev..

[95] Calabro F.J., Murty V.P., Jalbrzikowski M., Tervo-Clemmens B., Luna B. (2020). Development of Hippocampal-Prefrontal Cortex Interactions through Adolescence. Cereb Cortex.

[96] Keresztes A., Ngo C.T., Lindenberger U., Werkle-Bergner M., Newcombe N.S. (2018). Hippocampal Maturation Drives Memory from Generalization to Specificity. Trends Cogn. Sci..

[97] Kolk S.M., Rakic P. (2022). Development of prefrontal cortex. Neuropsychopharmacology.

[98] Yavas E., Gonzalez S., Fanselow M.S. (2019). Interactions between the hippocampus, prefrontal cortex, and amygdala support complex learning and memory. F1000Res.

[99] Roach L.A., Byrne M.K., Howard S.J., Johnstone S.J., Batterham M., Wright I.M.R., Okely A.D., de Groot R.H.M., van der Wurff I.S.M., Jones A.L. (2021). Effect of Omega-3 Supplementation on Self-Regulation in Typically Developing Preschool-Aged Children: Results of the Omega Kid Pilot Study-A Randomised, Double-Blind, Placebo-Controlled Trial. Nutrients.

[100] Osendarp S.J., Baghurst K.I., Bryan J., Calvaresi E., Hughes D., Hussaini M., Karyadi S.J., van Klinken B.J., van der Knaap H.C., Lukito W. (2007). Effect of a 12-mo micronutrient intervention on learning and memory in well-nourished and marginally nourished school-aged children: 2 parallel, randomized, placebo-controlled studies in Australia and Indonesia. Am. J. Clin. Nutr..

[101] Stephenson K., Callaghan-Gillespie M., Maleta K., Nkhoma M., George M., Park H.G., Lee R., Humphries-Cuff I., Lacombe R.J.S., Wegner D.R. (2022). Low linoleic acid foods with added DHA given to Malawian children with severe acute malnutrition improve cognition: A randomized, triple-blinded, controlled clinical trial. Am. J. Clin. Nutr..

[102] Oyen J., Kvestad I., Midtbo L.K., Graff I.E., Hysing M., Stormark K.M., Markhus M.W., Baste V., Froyland L., Koletzko B. (2018). Fatty fish intake and cognitive function: FINS-KIDS, a randomized controlled trial in preschool children. BMC Med..

[103] Al-Ghannami S.S., Al-Adawi S., Ghebremeskel K., Hussein I.S., Min Y., Jeyaseelan L., Al-Shammakhi S.M., Mabry R.M., Al-Oufi H.S. (2019). Randomized open-label trial of docosahexaenoic acid-enriched fish oil and fish meal on cognitive and behavioral functioning in Omani children. Nutrition.

[104] Keim S.A., Boone K.M., Klebanoff M.A., Turner A.N., Rausch J., Nelin M.A., Rogers L.K., Yeates K.O., Nelin L., Sheppard K.W. (2018). Effect of Docosahexaenoic Acid Supplementation vs Placebo on Developmental Outcomes of Toddlers Born Preterm: A Randomized Clinical Trial. JAMA Pediatr..

[105] Auestad N., Halter R., Hall R.T., Blatter M., Bogle M.L., Burks W., Erickson J.R., Fitzgerald K.M., Dobson V., Innis S.M. (2001). Growth and development in term infants fed long-chain polyunsaturated fatty acids: A double-masked, randomized, parallel, prospective, multivariate study. Pediatrics.

[106] Birch E.E., Garfield S., Hoffman D.R., Uauy R., Birch D.G. (2000). A randomized controlled trial of early dietary supply of long-chain polyunsaturated fatty acids and mental development in term infants. Dev. Med. Child Neurol..

[107] Mulder K.A., Elango R., Innis S.M. (2018). Fetal DHA inadequacy and the impact on child neurodevelopment: A follow-up of a randomised trial of maternal DHA supplementation in pregnancy. Br. J. Nutr..

[108] Fiske A., Holmboe K. (2019). Neural substrates of early executive function development. Dev. Rev..

[109] Bernhard W., Poets C.F., Franz A.R. (2019). Choline and choline-related nutrients in regular and preterm infant growth. Eur. J. Nutr..

[110] Nakazaki E., Yabuki Y., Izumi H., Shinoda Y., Watanabe F., Hishida Y., Kamimura A., Fukunaga K. (2019). Combined citicoline and docosahexaenoic acid treatment improves cognitive dysfunction following transient brain ischemia. J. Pharmacol. Sci..

[111] Scheltens P., Kamphuis P.J., Verhey F.R., Olde Rikkert M.G., Wurtman R.J., Wilkinson D., Twisk J.W., Kurz A. (2010). Efficacy of a medical food in mild Alzheimer’s disease: A randomized, controlled trial. Alzheimers Dement..

[112] Scheltens P., Twisk J.W., Blesa R., Scarpini E., von Arnim C.A., Bongers A., Harrison J., Swinkels S.H., Stam C.J., de Waal H. (2012). Efficacy of Souvenaid in mild Alzheimer’s disease: Results from a randomized, controlled trial. J. Alzheimers Dis..

[113] Wang D.D., Wu F., Ding L., Shi H.H., Xue C.H., Wang Y.M., Zhang T.T. (2021). Dietary n-3 PUFA Deficiency Increases Vulnerability to Scopolamine-Induced Cognitive Impairment in Male C57BL/6 Mice. J. Nutr..

[114] Aïd S., Vancassel S., Poumès-Ballihaut C., Chalon S., Guesnet P., Lavialle M. (2003). Effect of a diet-induced n-3 PUFA depletion on cholinergic parameters in the rat hippocampus. J. Lipid Res..

[115] Catalan J., Moriguchi T., Slotnick B., Murthy M., Greiner R.S., Salem N. (2002). Cognitive deficits in docosahexaenoic acid-deficient rats. Behav. Neurosci..

[116] Moreira J.D., Knorr L., Ganzella M., Thomazi A.P., de Souza C.G., de Souza D.G., Pitta C.F., Mello e Souza T., Wofchuk S., Elisabetsky E. (2010). Omega-3 fatty acids deprivation affects ontogeny of glutamatergic synapses in rats: Relevance for behavior alterations. Neurochem. Int..

[117] Dyall S.C., Michael G.J., Michael-Titus A.T. (2010). Omega-3 fatty acids reverse age-related decreases in nuclear receptors and increase neurogenesis in old rats. J. Neurosci. Res..

[118] Sidhu V.K., Huang B.X., Kim H. (2011). Effects of docosahexaneoic acid on mouse brain synaptic plasma membrane proteome analyzed by mass spectrometry and 16O/18O labeling. J. Proteome Res..

[119] Kodas E., Vancassel S., Lejeune B., Guilloteau D., Chalon S. (2002). Reversibility of n-3 fatty acid deficiency-induced changes in dopaminergic neurotransmission in rats: Critical role of developmental stage. J. Lipid Res..

[120] Chalon S. (2006). Omega-3 fatty acids and monoamine neurotransmission. Prostaglandins Leukot Essent Fat. Acids.

[121] de la Presa Owens S., Innis S.M. (1999). Docosahexaenoic and arachidonic acid prevent a decrease in dopaminergic and serotoninergic neurotransmitters in frontal cortex caused by a linoleic and alpha-linolenic acid deficient diet in formula-fed piglets. J. Nutr..

[122] Lands B., Bibus D., Stark K.D. (2018). Dynamic interactions of n-3 and n-6 fatty acid nutrients. Prostaglandins Leukot Essent Fat. Acids.

[123] Borsini A., Zunszain P.A., Thuret S., Pariante C.M. (2015). The role of inflammatory cytokines as key modulators of neurogenesis. Trends Neurosci..

[124] Lima I.V.d.A., Bastos L.F.S., Limborço-Filho M., Fiebich B.L., de Oliveira A.C.P. (2012). Role of Prostaglandins in Neuroinflammatory and Neurodegenerative Diseases. Mediat. Inflamm..

[125] Ern C., Frasheri I., Berger T., Kirchner H.G., Heym R., Hickel R., Folwaczny M. (2019). Effects of prostaglandin E(2) and D(2) on cell proliferation and osteogenic capacity of human mesenchymal stem cells. Prostaglandins Leukot Essent Fat. Acids.

[126] Lands B. (2008). A critique of paradoxes in current advice on dietary lipids. Prog. Lipid Res..

[127] Pestka J.J., Akbari P., Wierenga K.A., Bates M.A., Gilley K.N., Wagner J.G., Lewandowski R.P., Rajasinghe L.D., Chauhan P.S., Lock A.L. (2021). Omega-3 Polyunsaturated Fatty Acid Intervention Against Established Autoimmunity in a Murine Model of Toxicant-Triggered Lupus. Front. Immunol..

[128] Wierenga K.A., Harkema J.R., Pestka J.J. (2019). Lupus, Silica, and Dietary Omega-3 Fatty Acid Interventions. Toxicol. Pathol..

[129] Muller O., Krawinkel M. (2005). Malnutrition and health in developing countries. CMAJ.

